# Exploration of tissue-specific gene expression patterns underlying timing of breeding in contrasting temperature environments in a song bird

**DOI:** 10.1186/s12864-019-6043-0

**Published:** 2019-09-02

**Authors:** Veronika N. Laine, Irene Verhagen, A. Christa Mateman, Agata Pijl, Tony D. Williams, Phillip Gienapp, Kees van Oers, Marcel E. Visser

**Affiliations:** 1 Department of Animal Ecology, Netherlands Institute of Ecology (NIOO-KNAW), P.O. Box 50, 6700 AB Wageningen, The Netherlands; 2 Department of Biological Sciences, Simon Fraser University, Burnaby, Canada

**Keywords:** Transcriptomics, Seasonal timing, Aves, Selection line

## Abstract

**Background:**

Seasonal timing of breeding is a life history trait with major fitness consequences but the genetic basis of the physiological mechanism underlying it, and how gene expression is affected by date and temperature, is not well known. In order to study this, we measured patterns of gene expression over different time points in three different tissues of the hypothalamic-pituitary-gonadal-liver axis, and investigated specifically how temperature affects this axis during breeding. We studied female great tits (*Parus major*) from lines artificially selected for early and late timing of breeding that were housed in two contrasting temperature environments in climate-controlled aviaries. We collected hypothalamus, liver and ovary samples at three different time points (before and after onset of egg-laying). For each tissue, we sequenced whole transcriptomes of 12 pools (*n* = 3 females) to analyse gene expression.

**Results:**

Birds from the selection lines differed in expression especially for one gene with clear reproductive functions, zona pellucida glycoprotein 4 (*ZP4*), which has also been shown to be under selection in these lines. Genes were differentially expressed at different time points in all tissues and most of the differentially expressed genes between the two temperature treatments were found in the liver. We identified a set of hub genes from all the tissues which showed high association to hormonal functions, suggesting that they have a core function in timing of breeding. We also found ample differentially expressed genes with largely unknown functions in birds.

**Conclusions:**

We found differentially expressed genes associated with selection line and temperature treatment. Interestingly, the latter mainly in the liver suggesting that temperature effects on egg-laying date may happen down-stream in the physiological pathway. These findings, as well as our datasets, will further the knowledge of the mechanisms of tissue-specific avian seasonality in the future.

**Electronic supplementary material:**

The online version of this article (10.1186/s12864-019-6043-0) contains supplementary material, which is available to authorized users.

## Background

Over recent decades, environmental change (e.g. climate change) has resulted in phenological shifts of spring events across trophic levels [1–4]. In seasonally breeding birds, environmental change has the most profound effect on timing of breeding, i.e. timing of egg-laying, a life-history trait with major fitness consequences (e.g. [5–8]). As such, seasonal timing of breeding has been under directional selection towards earlier egg-laying [9–11]. In order to predict the responses to directional selection on timing of breeding via genetic changes, we need to understand both the novel and intensified selection pressures posed by environmental change on, as well as the genetic variation in, timing of breeding. Only those parts of the mechanisms underlying timing for which there is genetic variation can show a response to natural selection; these are the ‘wheels’ natural selection can turn [12]. Finding the genetic basis of timing of breeding is, however, complicated because there is a complex physiological mechanism underlying it, in which different organs and different environmental variables at different moments in time play a role [13].

**Fig. 1:**
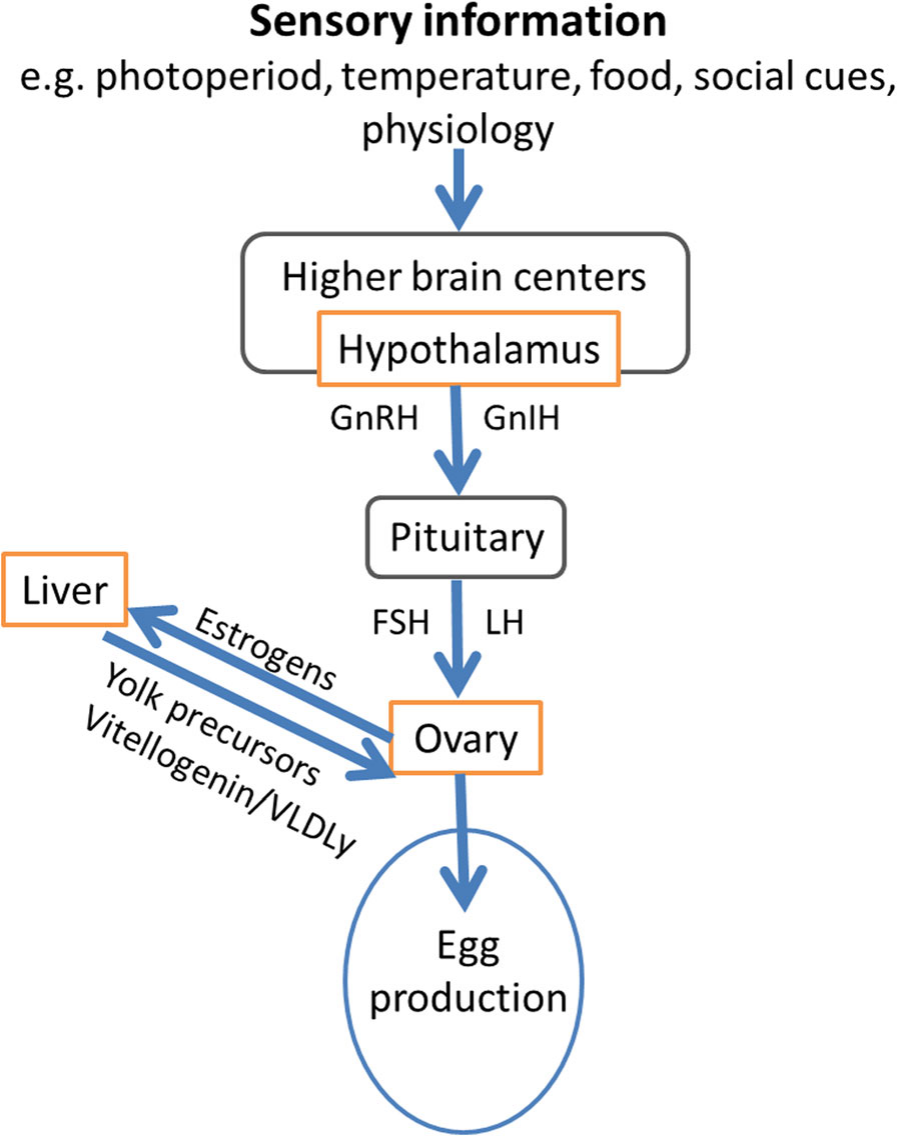
A schematic representation of the hypothalamic-pituitary-gonadal-liver axis in female birds (adapted from Williams 2012)

Photoperiod plays a main role in timing of breeding, as the yearly predictive increase in photoperiod in early spring provides precise information for birds to track the time of the year and stimulates the photoreceptors in the hypothalamus, which then send information along the photoperiodic signalling pathway [14, 15]. This pathway, in turn, triggers the synthesis and secretion of gonadotropin-releasing hormone (GnRH) in the hypothalamus, which marks the activation of the hypothalamic-pituitary-gonadal-liver (HPGL) axis (Fig. 1, [14, 15]), a key pathway underlying gonadal growth and maturation in anticipation of the breeding season and ultimately timing of breeding [16, 17].

While the function of photoperiod is clear in timing of breeding [16] and its signalling pathway prior to the activation of the HPGL axis is well-known at the molecular level [15, 18, 19], this remains largely elusive for temperature. We know, that it has a causal effect on [20, 21] and ‘fine-tunes’ timing of breeding [17, 22], that breeding time varies greatly between and within females from one year to the next depending on spring temperatures [23–25], and that the effect of temperature varies throughout spring and across latitudes [23, 25]. Under global warming, seasonal breeding birds could use temperature information to adequately advance their egg-laying period. However, this advancement might at some point become constrained by the lack of responsiveness to the HPGL axis to an increasing temperature. This is implied by the weak relationship between the development of the HPGL axis and the onset of egg-laying [26], suggesting that the way temperature acts on timing by-passes some major components of the reproductive system. However, it is unclear via which mechanism temperature is perceived and integrated [27]. Thus, *how* temperature affects seasonal timing of breeding and if this is only in the brain, like photoperiod, or also elsewhere in the HPGL axis.

As pointed out above, changing environments pose selection pressures on phenological traits such as timing of breeding, and a better understanding of the regulation of different parts of the reproductive axis by environmental cues and its molecular basis is hence imperative, especially in the context of adaptation to climate change. For this study we use the great tit (*Parus major*), which is a model species in ecology and evolution, due to its willingness to breed in nest boxes, short generation time and large broods, and wide distribution [28]. In addition, the study system of great tits, relying on caterpillars, which in turn rely on oak bud burst, is a well-known system [29] and showed different rates in shifts between trophic levels due to changing environments [6, 9, 30, 31]. Recently, a comprehensive molecular toolbox became available, including a well annotated reference genome [32], whole transcriptomes and methylomes from several tissues [32–34] and two SNP chips, 10 k and 650 k [35, 36], making the exploration of the (epi)genetic architecture of life-history traits possible [34, 37–40]. In addition to this toolbox, using the 650 k SNP chip, selection lines for early and late egg-laying were created using genomic selection, which is selection based on multi-marker genotypes rather than on the phenotype [21, 41]. Nestlings (the F_1_ generation) were taken from wild broods of which the mother was either an extremely early or extremely late breeder. These chicks were genotyped and, based on their “genomic breeding values” (GEBVs), individuals were selected for early and late line breeding pairs to produce the F_2_ generation in captivity (for details see [21, 41]). The F_3_ generation was then generated from the F_2_.

Here, making use of abovementioned tools, we measured overall gene expression levels by means of RNA-seq based expression profiling in three different tissues in great tit females housed in contrasting temperature treatments at three different time points related to egg-laying. As such, we explore time, temperature and tissue-specific gene expression patterns underlying timing of breeding. In order to identify molecular pathways likely to be involved in timing of breeding and the potential effect of temperature on these pathways, we performed functional gene enrichment analysis, network construction and hierarchal clustering of the RNA-seq datasets. In addition to exploring the molecular basis of seasonal breeding, our datasets and results will be an important starting point for future studies, especially on wild avian reproduction.

## Results

### Phenotypic results

The phenotypic results are described in detail in [21]. In short, we found no effect of either selection line, temperature treatment or their interaction (see “Experimental setup and samples” in materials and methods) on egg-laying dates (see “First breeding season” in materials and methods) and follicle size (see “Second breeding season” in materials and methods). However, follicles were significantly larger at time point 3 compared to time points 1 and 2.

### Sequencing and alignment

For the downstream analyses, we sequenced on average 18 ± 3 million (mean ± s.d.) single end reads in hypothalamus, 16 ± 2 million reads in liver and 15 ± 2 million reads in ovary and the overall alignment rate was on average 82.3% in hypothalamus, 79.8% in liver and 91.2% in ovary (Additional file 1: Table S1).

### Differentially expressed genes (DEGs)

When using the ‘regularized log transformation procedure’ (rld) transformed expression values from the DeSeq2 [42] package in the principal component anaylsis (PCA), we found that in the hypothalamus there was no clear clustering among time points, line or treatment (Additional file 18: Figure S1a). The PC1 explained 38% of the variance and that of PC2 is 22%. However, in liver and ovary the PC1 (with over 50% variation explained) clearly separated time point 3 samples from time points 1 and 2 (Additional file 18: Figure S1b&c, respectively). Taken together, the PCA analysis provided the first evidence of a clear distinction of gene expression profiles between different time points especially within liver and ovary in our dataset.

**Fig. 2:**
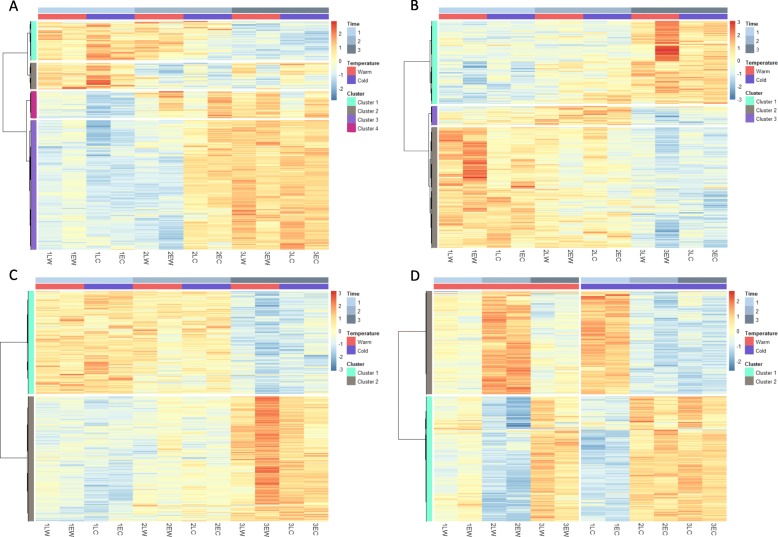
Heat map of genes that were significantly differentially expressed in the time point main models in hypothalamus (**a**), liver (**b**) and ovary (**c**) and in the time point x temperature interaction model in hypothalamus (**d**) (Note that in (**d**) samples were grouped based on temperature treatment, which differs from **a-c**). Genes were clustered by distances based on Pearson correlation coefficients in both figures. Lighter colours indicate lower differential expression; row Z- score scales from − 3 (dark blue) to 3 (dark orange)

In the differential gene expression analysis with DeSeq2, we found significant differences between time points in 491, 569 and 5175 transcripts in hypothalamus, liver and ovary, respectively (Table 1, Fig. 2; Additional files 3, 4, 5: Tables S3–S5 and Additional files 19, 20, 21: Figures S2-S4). We also did pairwise comparison with one time point to the two other time points and most of the expression differences occurred in time point 1 and 3 (Additional file 2: Table S2).

**Table 1:** The number of genes showing significant differential expression in the main effect models for the three tissues. Time point refers to contrasts between the three time points. Line refers to contrasts between the early and late selection line and temperature is the contrast between the warm and cold treatment

Tissue	Timepoint	Line	Temperature
Hypothalamus	491	26	5
Liver	569	10	30
Ovary	5175	46	2

There was a line effect in hypothalamus and ovary (Table 1). In the line main effect model for ovary one gene, the zona pellucida glycoprotein 4 (*ZP4*), clearly stood out having a strong differentiation between lines (Additional files 21 and 26: Figures S4 and S9, Additional file 5: Table S5).

Most of the DEGs between warm and cold treatments were found in liver while, interestingly, the hypothalamus showed a significant interaction effect between time point and temperature forming two clear clusters of upregulated genes (Fig. 2d, Additional file 2: Table S2). The pools from the warm condition were shifted between time points compared to the cold treatment (Fig. 2d). For the other interaction models there were 0 to 14 differentially expressed genes in all of the tissues (Additional file 2: Table S2).

### Hierarchical clustering of DEGs and GO enrichment analysis

We used hierarchical clustering of the DEGs to determine clusters of genes that changed through time in a similar way. We identified four, three and two clusters in hypothalamus, liver and ovary time point models respectively, and two groups in the hypothalamus time point-temperature interaction model (Fig. 2, Additional files 22, 23, 24, 25: Figures S5-S8 and Additional files 3, 4, 5: Tables S3-S5). Each cluster had a particular expression profile over time (and temperature in the hypothalamus interaction model). In most clusters there was a linear increase or decrease of expression towards time point 3, but there were one cluster in hypothalamus (clusters 2) and one cluster (cluster 3) in liver which showed relatively higher/lower expression at time point 2 compared to time points 1 and 3.

A functional enrichment analysis was possible for the time point main-effect models for all of the tissues and also for the time point-temperature interaction model for hypothalamus when the significance level was set to *p* < 0.05 for DEGs. Enrichment analysis for the main effect model in hypothalamus showed that in genes that were upregulated in time point 1 (cluster 1) 11 different GO categories and KEGG pathways were overrepresented. These were related especially to circadian rhythm related GO terms and pathways (Additional file 6: Table S6). Genes that had increased expression towards time point 3 (clusters 3 and 4) had 45 different GO terms and KEGG pathways overrepresented and from these especially GABA activity and other neuronal function related GO groups were significantly enriched. Cluster 2 (low expression at time point 2) had one GO category overrepresented.

In the interaction model between time point and temperature for hypothalamus, there were two gene clusters. Cluster 1 genes contained upregulated genes at time point 3 in both temperatures but the expression pattern in time points 1 and 2 differed between warm and cold treatments. In Cluster 2 the expression pattern was the opposite; upregulated genes at time points 1 and 2 compared to time point 3 and differing patterns between temperature treatments in time point 3. The genes and GO groups in cluster 1 (242 functional terms) were similar to the main model results with functions related to neuronal activity and the GABA pathway. However, the upregulated genes in cluster 2 (323 functional terms) were related to ribosomal, mitochondrial and ATP related metabolic functions (Additional file 7: Table S7).

In liver, there were 130 GO terms and KEGG pathways enriched in genes that were upregulated in time point 1 (cluster 2). These terms and pathways were related to immunological functions, hormone responses and insulin response. Genes upregulated at time point 2 (cluster 3) were linked to two GO terms: carbon-nitrogen lyase activity and oxidoreductase activity. In time point 3 (cluster 1) 32 GO groups and KEGG pathways were enriched which were especially related to protein processing and amino acid response (Additional file 8: Table S8). Furthermore, egg-laying related genes, cathepsin E-A-like gene (LOC107205210, *CTSEAL*), vitellogenin 2 (*VTG2*; LOC107208431 and LOC107208432) and apovitellenin 1 (*APOV1,* LOC107200088) were expressed at this time point (Additional file 27: Figure S10 and Additional file 4: Table S4). The expression level increase of *VTG2* and *APOV1* had fold change of nine and *CTSEA*L fold change of 7 from time point 1 to time point 3 where early line showed larger increase.

In ovary, the genes that were upregulated at time point 1 (cluster 1 with 130 functional groups) were related to cell cycle, chromosome functions and spindle formation (Additional file 9: Table S9). Five bird-specific egg related genes; *VTG2*, ovalbumin (*OVAL*; LOC107215075), ovalbumin-related protein Y (*OVALY*, LOC107214443), lamin-L(III)-like (*LMINA*; LOC107209405) and avidin (*AVD*; LOC107198337), were expressed at time point 1. In time point 3 (cluster 2 with 803 functional groups) genes were related to morphogenesis and development. The “egg-laying gene” *APOV1* was expressed at time point 3 and also bird specific major histocompatibility complex class II beta chain (*BLB2*; LOC107199337) gene (Additional file 5: Table S5).

To explore the tissue specific circadian gene activity, we compared our DEGs to the genes from the super pathway ‘BMAL1-CLOCK, NPAS2 activates circadian gene expression’ from Path Cards, a pathway unification database (http://pathcards.genecards.org; [43]), which lists 86 genes that activate the circadian gene expression pathway. In total, we found 41 genes of this pathway that were significantly differentially expressed between time points or in interaction with temperature in hypothalamus. Most of these circadian genes were found in ovary (28 genes) (Additional file 10: Table S10).

### Weighed correlation network and hub gene analysis

To investigate the patterns of co-expression among transcripts, we analysed the rld transformed data using weighed correlation network analysis (WGCNA) [44]. We constructed five, nine and six co-expression modules for hypothalamus, liver and ovary, respectively that were significantly associated with the treatments (Table 2). There were modules that were significantly correlated with every treatment in liver but in hypothalamus and ovary none of the modules correlated with the temperature (Table 2, Additional files 29, 30, 31: Figures S12-S14). There was an overlap with the genes between modules and DEGs where most of the overlap was with the time point model in every tissue type and also the interaction of time and temperature in hypothalamus suggesting time point being the most driving effect of co-expression in our samples (Table 3). Details of the transcripts belonging to the modules are provided in Additional files 11, 12, 13: Tables S11–13.

**Table 2:** Summary of gene modules identified with weighted correlation network analysis (WGCNA). Only showing modules with significant correlation with the treatments. Network/module is number of genes found by STRING analysis out of the whole set of module genes that passed the selection threshold (ModuleMembership > 0.8 and treatment *p*-value > 0.05). Top hub genes were chosen based on high modular membership (kME) value and highest degree in PPI network

Tissue	Module color	Number of genes	Most significant correlation	Network/module	Hub gene symbol	Gene name
Hypothalamus						
	Brown	1668	Time point	117/130	*ADCY2*	adenylate cyclase 2
	Turquoise	5631	Time point	395/493	*HSPA8*	heat shock protein family A (Hsp70) member 8
	Blue	5068	Time point	784/911	*MAPK1*	mitogen-activated protein kinase 1
	Green	1022	Time point	65/72	*EPRS*	glutamyl-prolyl-tRNA synthetase
	Yellow	1031	Line	55/58		
Liver						
	Pink	714	Temperature	41/49		
	Turquoise	3149	Time point	202/264	*GART*	phosphoribosylglycinamide formyltransferase…
	Magenta	537	Time point	30/31	*SNAP25*	synaptosomal-associated protein 25
	Red	901	Time point	32/39		
	Green	951	Time point	39/46	*HSPA4*	heat shock protein family A (Hsp70) member 4
	Blue	3115	Time point	263/289	*PTPRC*	protein tyrosine phosphatase, receptor type C
	Purple	434	Time point	21/25		
	Salmon	363	Line	25/34	*POLR3B*	RNA polymerase III subunit B
	Midnightblue	160	Line	6/13		
Ovary						
	Yellow	2229	Time point	147/174	*HSP90AA1*	heat shock protein 90 alpha family class A member 1
	Turquoise	6579	Time point	913/1372	*ACLY*	ATP citrate lyase
	Blue	5573	Time point	1722/2252	*SRC*	SRC proto-oncogene, non-receptor tyrosine kinase
	Brown	3093	Time point	557/658	*AKT1*	AKT serine/threonine kinase 1
	Cyan	123	Line	5/15		
	Midnightblue	102	Line	3/3

**Table 3:** The number of significantly differentially expressed genes from time point, temperature and line models overlapping with the members of gene modules from WGCNA (see Table 2). Only showing modules with significant correlation with the treatments

Tissue	Module color	Total	Time	Temperature	Line
Hypothalamus					
	Brown	96	57	0	6
	Turquoise	770	78	1	1
	Blue	1070	280	1	0
	Green	41	32	0	6
	Yellow	44	21	0	4
Liver					
	Pink	10	5	5	0
	Turquoise	185	176	4	0
	Magenta	8	7	0	0
	Red	83	71	10	0
	Green	53	48	3	0
	Blue	214	205	4	1
	Purple	16	16	0	0
	Salmon	7	2	0	5
	Midnightblue	1	1	0	0
Ovary					
	Yellow	184	172	0	7
	Turquoise	1996	1967	0	17
	Blue	2242	2233	1	2
	Brown	579	576	0	2
	Cyan	7	6	0	1
	Midnightblue	3	3	0	0

**Fig. 3:**
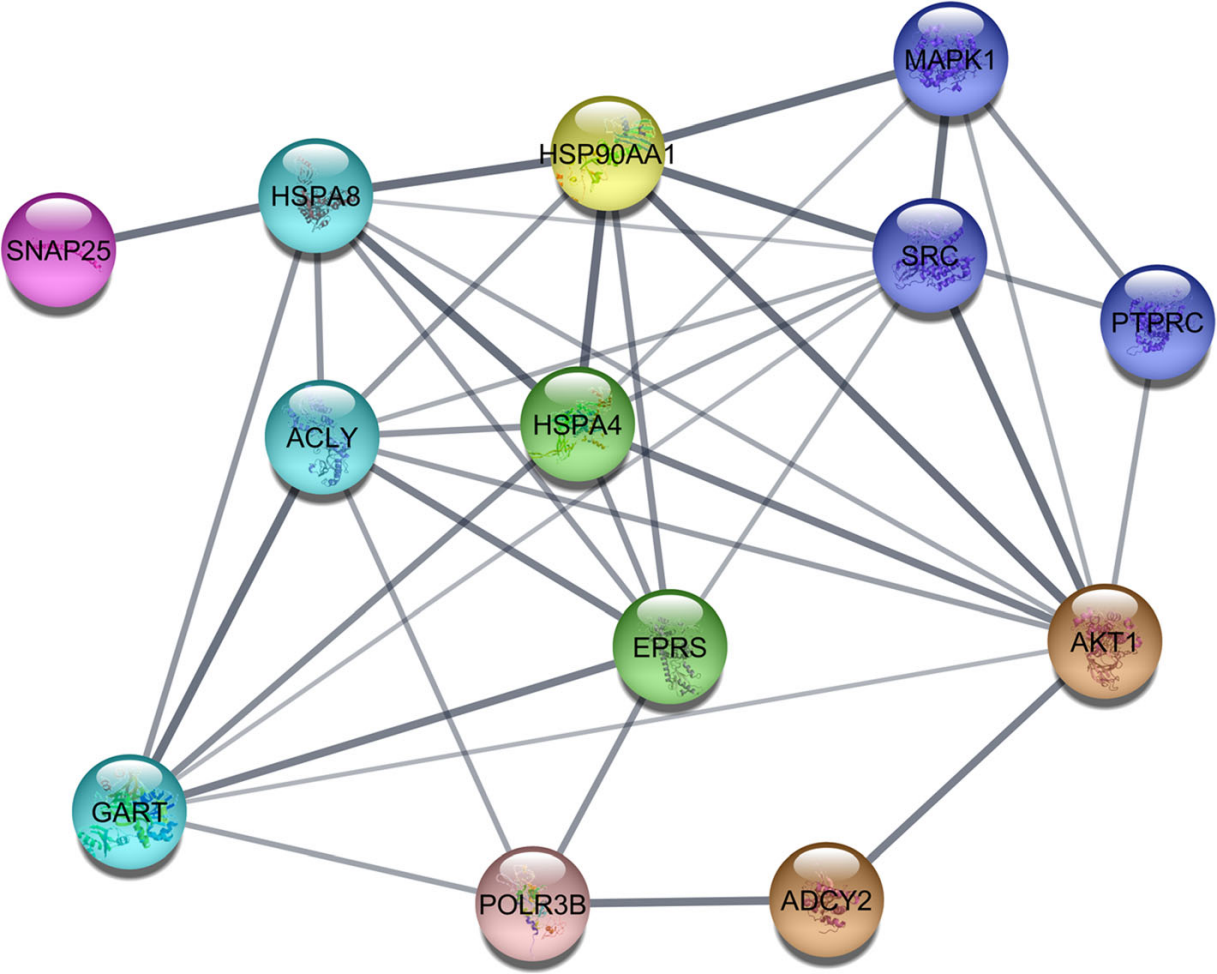
The PPI network of the “real” hub genes from all tissues combined. The line thickness indicates the node connection score; thicker line means more evidence for the connection from existing research (experimental, co-expression, database). Colours correspond with modules from Tables 2 and 3

We could determine 13 “real” hub genes out of 21 modules based on combination of co-expression and PPI network connections (Table 2). The network analysis of “real” hub genes from each module significantly associated with the treatments showed that all the genes (Additional files 14, 15, 16: Tables S14–16), were in the same PPI network with all of them belonging to molecule binding GO term and most significant pathways were estrogen signalling and progesterone-mediated oocyte maturation pathways (Fig. 3 and Additional file 17: Table S17).

## Discussion

We measured gene expression levels in 12 pools of three female great tits each, from two lines selected for early and late egg-laying, which were kept at two contrasting temperature treatments, and were sampled at three time points across the breeding season. Most of the DEGs varied between time points 1 (well before egg-laying) and 3 (at the time of egg-laying). Gene expression levels of females from both lines and temperature treatments were following similar patterns in ovary and liver. In hypothalamus, however, we found a significant interaction between time point and temperature, which could indicate that temperature affects the timing of certain gene expression levels mainly in the brain. We found no effect of temperature on either egg-laying date in the first breeding season or follicle size in the second breeding season (see “Downstream regulation of timing of breeding” below). Many of the highly DEGs had an unknown function; either being non-coding RNA or the gene has an unknown function especially in birds. Furthermore, in every tissue we identified hub genes that may play a central role in timing of reproduction in great tits.

### Limitations of the data

Due to the small sample size for each time point in this study, the statistical analysis likely suffered from low power to detect differences between time points, temperature and line. We used pooled data without any replication and especially the interaction in hypothalamus would have benefitted of having individual level expression data with replication. Unfortunately, it is not possible to obtain tissue samples from the same individual in every time point to see how the expression patterns change in one individual. In great tits it has been shown that there is some genetic variation both in the onset and termination of egg-laying, and in the underlying mechanisms, and sometimes there is also an interaction with temperature [45, 46]. Some families of birds are able to respond more quickly than others to the increasing temperature, which leads to differences in timing of breeding between families [46–50]. However, in our case we tried to minimize the relatedness within the pools and both lines were grouped in a similar way with regards to the temperature so relatedness might not play an important role for these results. We are thus positive that our results give a comprehensive overview of the different genes being expressed during seasonal timing as the PCA, differential expression and WGCNA give similar results. In addition, the results found also match with those described in the literature. In future studies, however, it is essential to confirm these results at the individual level and have additional time points before time point 1, as used here, in order to pin point the exact moment when preparing for breeding starts (i.e. activation of the HPGL axis). Furthermore, having gene expression levels in both the ovary tissue and follicular tissue could help us to recognise specific ovarian and/or follicular functions. Also, the addition of other tissues would help building the whole network of interacting genes [51].

### Downstream regulation of timing of breeding?

Although gene expression levels in the hypothalamus seem to be affected by temperature, this does not *directly* lead to earlier egg-laying, because we found no effect of temperature on either egg-laying date (first breeding season) or follicle size (second breeding season). Our data are in line with the hypothesis that downstream processes in the liver and ovary play a more important role in the fine-tuning of egg-laying date than hypothalamic processes [22, 52–54]. In this sense, the absence of an effect on egg-laying date of temperature is informative on where in the neuro-endocrine cascade fine-tuning occurs, rather than that it hampers new insights.

### Time point effects

#### Time point 1

At time point 1, the genes expressed in the hypothalamus were related to circadian rhythm and photoperiodism. In fact, in every time point and every tissue, also in the interaction model in hypothalamus, several genes involved in circadian rhythms were differentially expressed. In addition to the HPGL axis, the role of the circadian clock in annual cycles has been suggested for some time [55, 56]. The circadian phase at which light affects the photoreceptive elements causes reproductive changes [57]. The core of the avian circadian system is located in the pineal gland [58]. This core clock acts as a master regulator of the rhythms of peripheral tissues. In birds a rhythmic expression of the clock genes has been identified in the mediobasal hypothalamus, suggesting that this structure contains the circadian pacemaker associated with photoperiodic time measurement [15, 59].

Interestingly, there was not much overlap in circadian genes between tissues and also between the two models (main effect and interaction) in hypothalamus. The more downstream tissues (i.e. ovary and liver) also possess their own circadian clockworks and entrain their tissue-specific rhythms through their own, the core or both outputs of the circadian system [60–62]. Especially the circadian clocks in the ovary may play a role in the timing of ovulation [61–63]. The circadian genes from the hypothalamus main model were mostly related to regulation and entrainment of the circadian rhythm. In the hypothalamus genes related to activin receptor signalling pathway were also upregulated at time point 1. Activin which is produced by gonads but also in extragonadal tissues, can enhance FSH biosynthesis in the pituitary gland and in hypothalamus activin stimulates GnRH release and thereby affects the levels of FSH and LH [64–67].

In liver, eight differentially expressed molecular clock related genes were mainly expressed at time point 1 with their majority being circadian regulators of gene expression (nuclear receptor subfamily 1 group D member 1, *NR1D1;* neuronal PAS domain protein 2, *NPAS2;* period circadian clock 2*, PER2;* period circadian clock 3*, PER3* and basic helix-loop-helix family, member e41, *BHLHE41*). In birds, changes in circadian gene expression in liver has been linked to alteration in the seasonal state [68]. However, timing of the circadian clock in liver is often controlled by feeding rather than by the core clock system the brain [69, 70]. At time point 1 also the estrogen signalling pathway and hormone stimulus related GO terms were enriched suggesting that the liver could be processing hormonal signals from the ovary in order to start vitellogenesis later in the season. In addition, at time point 1 there were also genes upregulated that were belonging to immunological and insulin related functions. Both adaptive and innate immune responses produced by liver have been found in chicken ovaries, and these systems function to protect against colonization and infection by microbial pathogens, as well as to maintain normal functions of the ovary [71]. Insulin is suggested to be one of the key regulators of reproductive function by having an effect on GnRH/LH secretion [72].

We found that the ovary exhibited the most DEGs and co-expressed genes. In the pools at time point 1 ovary was expressing genes that were related to cell cycle, mitosis and meiosis suggesting that it already started with the ovarian maturation, along with follicle development [73, 74]. For example, the expression of the genes important for follicular development such as the transforming growth factor beta (TGF-β) superfamily (such as growth differentiation factor-9, *GDF9* and bone morphogenetic protein 15, *BMP15*) [75, 76] and other genes such as forkhead box L2 (*FOXL2*) and NOBOX oogenesis homeobox (*NOBOX*) [74] were already high so it seems that great tits start folliculogenesis six weeks before laying the first eggs. There is an intermediate pre-vitellogenic follicle development phase in chicken which resides between the slow stage which is the development of primordial follicles and can last several months and the rapid follicle/rapid yolk development growth stage which can happen just few days before laying the first eggs [71]. During this intermediate phase small amount of lipoprotein rich white yolk are incorporated to the follicles increasing slightly their size and some of them are selected to final maturation stage [71]. Because vitellogenesis in liver and the increased expression of LH receptors in ovary happens at time point 3, our birds might indeed be in the pre-vitellogenic phase at time point 1 (and also 2), as shown by follicular measurements in the same females [54].

#### Time point 2

Many of the DEG clusters from the time point 1 were also upregulated at time point 2 such as the circadian and activin related genes in hypothalamus and ovary’s cell cycle related genes. In hypothalamus there was also a cluster of genes that were starting to be expressed at time point 2 and continued to be highly expressed at time point 3 as well. These genes were related to female reproduction such as the genes progesterone receptor (*PGR*) and prolactin receptor (*PRLR*, see below). There were also genes part of angiogenesis and one of them being fibroblast growth factor (*FGF1*) which has also been shown to be linked to egg fecundity in chicken albeit from the bone RNA samples [77].

In liver there was a specific upregulated gene cluster on time point 2. These genes were related to oxidoreductase and carbon-nitrogen lyase activity which do not have known function in reproduction. Both GO groups shared one gene, the aldo-keto reductase family 1 member B10 (*AKR1B10*) which is known to be part in detoxifying compounds under oxidative stress conditions and it has also been shown in humans that aldo–keto reductases are part of steroid hormone action and nuclear receptor signalling [78]. Oxidoreductase related functions continued being important as well at time point 3 where also amino acid metabolism and protein processing related GO groups were associated in which both oxidoreductase enzymes are important factors.

#### Time point 3

In time point 3 in hypothalamus the upregulated genes were related to many neuronal function groups but also to GABA receptor functions. GABA, the main inhibitory neurotransmitter, and glutamate, the main stimulatory neurotransmitter, set a level of sensitivity in the hypothalamus that decreases or increases the likelihood that GnRH will be synthesized or released based on the reproduction status of the females [79]. Other HPGL-axis genes that are known to be expressed in hypothalamus such as gonadotropin-releasing hormone 1 (*GnRH1*) was not expressed in our hypothalamus samples. However, *GnIH* (but annotated as neuropeptide VF precursor, *NPVF* in great tit), iodothyronine deiodinase 2 (*DIO2*) and thyroid stimulating hormone receptor (*TSHR*) were active in hypothalamus and from these *TSHR* was especially expressed on time point 3 indicating HPG cascade going towards egg production [59].

At time point 3 in liver in addition to above mentioned metabolism and protein processing functional groups, vitellogenesis related genes were upregulated such as *VTG2* and *APOV1* which also showed line differences in expression levels where early lines had higher expression especially in the early-warm condition at this time point. Furthermore, cathepsin E-A-like gene (LOC107205210, *CTSEAL*) was upregulated at time point 3, which has been shown to be over-expressed during vitellogenesis in chicken liver and is regulated by estrogen [80, 81]. Next to *CTSEAL* in the great tit genome is bestrophin 3 (*BEST3*) which was also upregulated at time point 3. *BEST3* is an important gene in chloride channel activity but there is no known function in regards to reproduction. The similar expression pattern between *BEST3* and *CTSEAL* and their closeness in the genome suggests that they might be co-regulated but it is unclear in the current study if mRNA from *BEST3* used in the liver in the end. It is known that mRNA goes through several regulatory processes after it is made and this is often seen when comparing the expression levels from transcriptomes and proteomes [82, 83]. In addition to *BEST3,* we found additional genes from every tissue that have unknown function in bird reproduction. There were also transcripts that are annotated as ncRNA by the NCBI. This type of RNA has been shown to be important in eukaryotic gene regulation and also in hormonal pathways and meiosis during reproduction [84]. Furthermore, there is evidence that miRNAs are differentially expressed in the ovary from sexually immature versus mature chickens, and in developing ovarian follicles relative to the stage of maturation [85].

Most of the circadian genes were expressed in the ovary and especially at time point 3 supporting the idea that these genes are important in starting the ovulation in birds [62, 63]. At time point 1 the two period genes, *PER2* and *PER3*, were upregulated. In poultry these two have been linked to preovulatory follicle expression [61]. In general, it is suggested that expression of ovarian circadian clock genes may be influenced by the increase of LH which may be a mechanistic link for communicating circadian timing information from the core clock in the brain to the ovary [61, 86]. The receptors for FSH and LH (follicle-stimulating hormone receptor *FSHR*/LOC107202460 and lutropin-choriogonadotropic hormone receptor, *LHCGR*/LOC107201154) were expressed in ovary and especially the expression of *LHCGR* increased towards time point 3 suggesting increased LH activity in our ovary samples. In birds the increased expression of LH receptors in ovary starts the final follicle maturation [87]. In addition to of the circadian genes, many of the upregulated genes were also related to developmental and morphogenesis GO groups and pathways. Interestingly, the mitogen-activated protein kinase (MAPK) signalling pathway was active at this time point as well. MAPK is proposed to inhibit *FSHR* transcription and is part of the cascade where pre-hierarchal follicles are selected into the preovulatory hierarchy [88] which is important at the rapid follicle development stage.

### Temperature and line effects

In the hypothalamus gene expression was affected by the interaction between time point and temperature. However, due to limitations of the dataset the results should be treated as suggestive. Circadian genes were mostly expressed in time point 3 but there was a set of five circadian genes that were expressed at time point 1 which were mostly related to ubiquitination. Mutation in ubiquitin related genes can cause either elongation or shortening of the endogenous circadian period (tau) [89]. Interestingly, while photoperiod, nutrient and redox status can entrain the clock [60], temperature can affect the endogenous circadian period in great tits [90]. Furthermore, in the interaction model in hypothalamus there were circadian genes that are regarded as the core genes in the circadian rhythm pathway such as clock circadian regulator (*CLOCK*), *PER2* and RAR related orphan receptor A (*RORA*) which also have pleiotropic effects to many metabolic processes [60, 91].

In general, the interaction model had two gene clusters that showed distinctive patterns. The genes that were upregulated more during time point 1 and 2 in cold and warm environments, respectively were associated with metabolic-related terms and pathways such as ATP, NADH and ribosomal metabolic processes. The molecular clock constantly receives feedback from the metabolic signals in the cells [60, 92] and can affect metabolism of the organism and is also controlled by metabolic pathways. The terms related to the second cluster which had genes upregulated more at time point 3 were similar to main effect time point model in hypothalamus by having the GABA pathways but also the circadian related terms. However, in this cluster there were also dopaminergic synapse pathway related genes upregulated. Dopamine together with prolactin influences the HPG axis primarily at the level of the hypothalamus and pituitary, by regulating the release of the gonadotropic hormones [93, 94]. *PRLR* was indeed also upregulated at time point 3 in our samples suggesting that both dopamine and prolactin were active in hypothalamus.

In contrast to the hypothalamus, no convincing effect of an interaction between time point and temperature (or just temperature alone) was found in liver and ovary, which was not surprising as no difference in egg-laying was observed between the temperature treatments. Liver had 30 differentially expressed genes between the temperatures and it was the only tissue with a co-expression module associated with temperature. However, no GO enrichment analysis could be conducted with the genes and hub gene was not found in the module.

All the tissues showed some line differences in gene expression but in ovary one gene was highly differentially expressed. This was zona pellucida sperm-binding protein 4 (*ZP4*) which had a two to three times higher expression in early line compared to late line. It also appears in the co-expression results and is also under selection in these selection line birds [21]. *ZP4* is one of the genes responsible to making the zona pellucida (in mammals) or vitelline envelope (in fish, amphibians and birds), a glycoprotein layer surrounding oocytes [95]. The zona pellucida mediates sperm–egg interaction, provides a post-fertilization block to polyspermy, and protects the embryo prior to implantation [96]. In our selection line birds it is not known what role this gene plays between the lines.

### Real hub genes for every tissue

All the “real” hub genes that shared high interaction both in the co-expression and the PPI networks were all transcribing binding molecules and they were all in the same final PPI network. Six genes were found in the estrogen signalling pathway (three from hypothalamus; *MAPK1, HSPA8, ADCY2* and three from ovary; *AKT1, HSP90AA1, SRC*). In addition, MAPK pathway being important in the ovary, *MAPK1* is estrogen activated in the brain and is important in female sexual behaviour [97]. Both *MAPK1* and *HSPA8* have been found to be differentially expressed in hypothalamus during spring migration in black-headed buntings (*Emberiza melanocephala*) [98]. *ADCYC2* in hypothalamus and *SNAP25* in liver are important genes in insulin secretion and four genes are important in temperature detection (two in hypothalamus: *MAPK1, HSPA8* and two in ovary: *AKT1, HSP90AA1*). In addition to estrogen signalling pathway, other hormonal pathways related to reproduction were associated with these hub genes such as progesterone, thyroid, prolactin and oxytocin binding/signalling pathways suggesting that our hub genes are important in female reproduction.

## Conclusions

We generated comprehensive RNA expression data from a set of three tissues important in the neuro-endocrine cascade underlying avian seasonal timing of breeding, from three different time points and from two temperature treatments and two selection lines for breeding time. Time was the strongest driving variable in our dataset, as we would expect, but there was an interesting interaction between time and temperature in hypothalamus which should be studied more intensively in the future studies. It could be possible that gene expression in the brain is affected by temperature, perhaps through changes in expression of genes involved in the circadian clock which affect the sensitivity to photoperiod. However, because laying dates were not directly affected by temperature, the effect of temperature on timing of breeding is likely fine-tuned downstream in the reproductive axis, i.e. the liver and/or the ovary, rather than upstream, in the hypothalamus. These findings, as well as our datasets, will further the knowledge of the mechanisms of tissue-specific avian seasonality in the future.

## Methods

### Experimental setup and samples

A detailed description of the experimental setup and sampling is described in [21]. In short, 36 great tit pairs (18 *early line* and 18 *late line* pairs) originating from the second generation (F_2_) of lines artificially selected for early and a late timing of breeding (for details see [21, 41]), were housed in 36 climate-controlled aviaries (2 m × 2 m × 2.25 m) at the Netherlands Institute of Ecology (NIOO-KNAW). Birds were subjected to a photoperiod mimicking the natural photoperiod and to two contrasting environments mimicking a cold spring (2013) and a warm spring (2014) in the Netherlands (Additional file 32: Figures S15). Temperatures changed every hour to follow as closely as possible the observed hourly temperatures in these years. The combination of selection line and temperature environment resulted in four groups: ‘early-warm’, ‘early-cold’, ‘late-warm’ and ‘late-cold’. Birds were fed ad libitum with a constant daily amount, had water available for drinking and bathing and their welfare were assessed twice a day by animal caretakers [46]. The pairs were used in two consecutive breeding seasons within one year (see [54] for details); a first breeding season in spring and a second breeding season in autumn, after the birds went to a period of short-day length and low temperatures (see below).

#### First breeding season

In the first breeding season, initiated on 4 January 2016, the four groups were kept in pairs in the climate-controlled aviaries during spring. Nesting material (moss and hair) was provided from the second week of March onwards. Females could choose between three nest boxes of which two were accessible from the outside to minimalize disturbance. Females initiated nest building and subsequent egg-laying, which were recorded together with other reproductive traits (e.g. clutch size). In addition, both sexes were blood sampled bi-weekly throughout the breeding season as part of another study [99].

#### Second breeding season

**Fig. 4:**
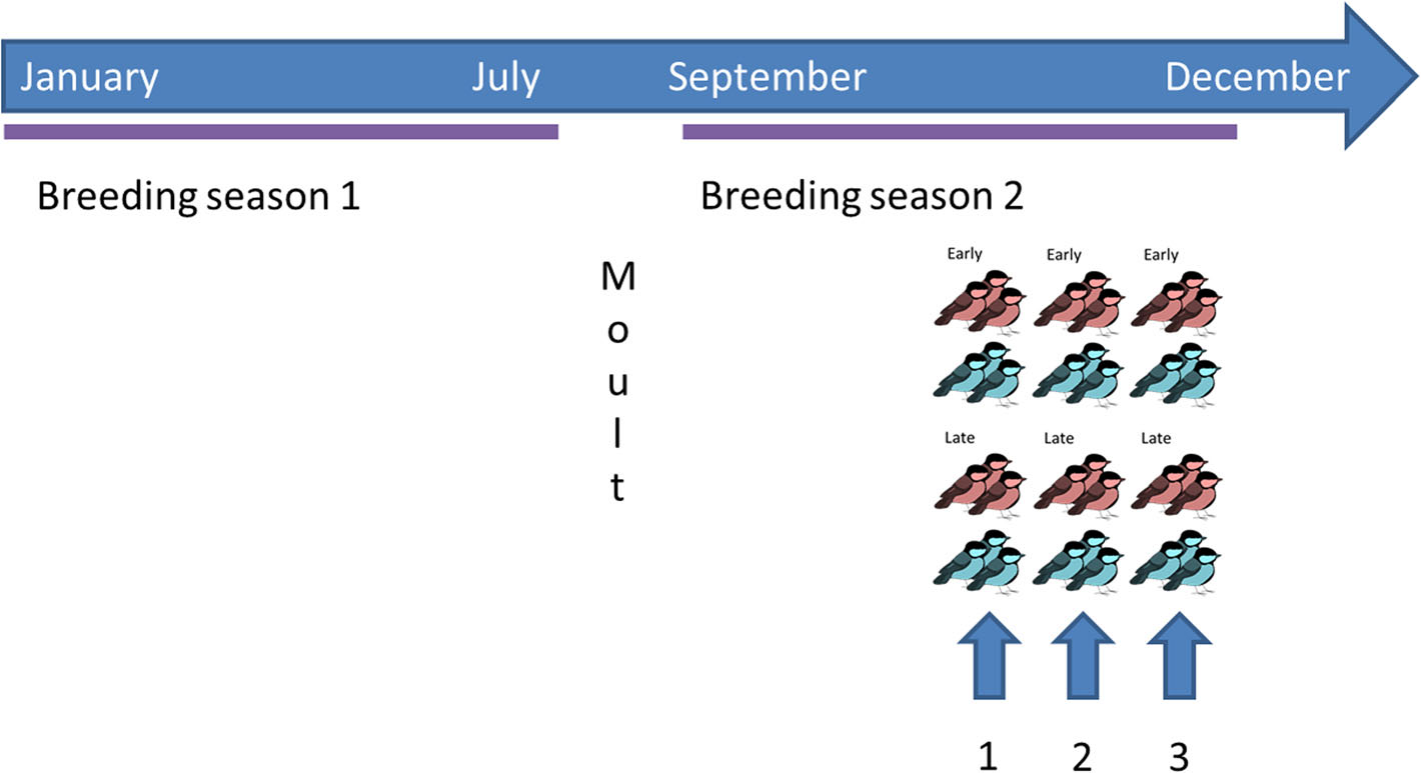
Visualization of the experiments through which the F2 females (representing all four selection line x treatment combinations) in this study went. Females subjected to the warm and cold treatment are indicated in red and blue, respectively. The blue arrows indicate the three time points on which the tissues were collected: time point 1 when day length exceeds 11 h, time point 2 when nest building occurs in the first breeding season and time point 3 when egg-laying was initiated in the first breeding season by 25% of the females

After the first breeding season, when birds were well on their way moulting (~mid-July), days were shortened to 9 L:15D and temperatures decreased to 10 °C for seven weeks to make the birds photosensitive and temperature sensitive again. From September onwards, the pairs were subjected to the same photoperiod and temperature regimes again as in their first breeding season, to initiate their second breeding season. Four females were replaced with a sister, because they did not initiate egg-laying in the first breeding season. Females showed similar phenotypic responses in the first and the second breeding season (a significant correlation between lay date in the first breeding season and ovary size at time of sacrifice in the second breeding season; [54]). Therefore, pairs were divided in three groups (*n* = 12 pairs per group) as such that the egg-laying date distribution (recorded in the first breeding season) were similar per group. Every group was sacrificed at a different time point (see “Tissue collection and preparation”, Fig. 4).

#### Tissue collection and preparation

Three time points throughout the second breeding season were chosen, based on the reproductive behaviour from the first breeding season: (1) October 7 (resembling March 7) when photoperiod exceeded 11 h [100], (2) October 28 (resembling March 30) when nest building occurred in the first breeding season, but prior to egg-laying and (3) November 18 (or April 20) when about 25% of the females in 2015 had initiated egg-laying in the first breeding season. We sacrificed one group (both males and females, but we focus on the females in this study) per time point (see [54] for details, Fig. 4). In short, birds were caught per pair between 9 and 12 AM from the aviaries, taken to the operation room and deeply anaesthetized with Isoflurane (IsoFlo, Zoetis, Kalamazoo, Michigan) using breathing mask during which a blood sample was also taken, followed by decapitation. Tissues, including brain, gonads and liver were dissected and stored in − 80 °C until further processing. At a later stage, the hypothalamus was isolated from the rest of the brain and, until further processing, stored in − 80 °C.

#### RNA extraction and sequencing

From hypothalamus, ovary and liver, RNA was isolated by Trizol extraction (see [54] for details). We pooled RNA of three females per time/line/treatment group, resulting in a total of 12 pools (Fig. 4). The library preparation and sequencing were performed at Baseclear, Leiden, The Netherlands. Libraries were made using the Illumina TruSeq strand-specific mRNA method ((Illumina, San Diego, CA, USA). We used one lane of Illumina HiSeq 2500 (single-end 50 bp) for 12 pools. About 192 million single-end reads of 50 bp were generated for liver, 219 million reads for hypothalamus and 181 million reads for ovary.

### RNA-seq analysis

#### Sequence data processing and differential gene expression analysis

Filtering of low quality reads was conducted at Baseclear by removing PhiX and adaptor sequences. The trimmed reads were mapped to the *Parus major* reference genome build 1.1. (https://www.ncbi.nlm.nih.gov/assembly/GCF_001522545.2) using Hisat2 v2.1.0 [101] with default parameters. Transcript assembly was done using Cufflinks v2.2.1 [102], with default parameter settings and based on the *Parus major* annotation release 101 in NCBI (https://www.ncbi.nlm.nih.gov/genome/annotation_euk/Parus_major/101/). The obtained annotations were merged using cuffmerge. Unique reads that mapped to merged transcripts were counted using HTSeq v0.9.1 [103].

All analyses were performed and figures made in R v.3.4.4. Clustering of the samples was done using Principal Component Analysis (PCA) using the ‘regularized log transformation procedure’ (rld) transformed expression values in order to diminish the number of variables and summarize the data. Differential expression of genes (DEG) between different time points, line and temperature were performed with DeSeq2 v3.6 [42] using the standard DeSeq2 protocol and Likelihood Ratio Test (LRT). LRT is useful for testing multiple terms at once compared to the default Wald test. The test examines two models, a full model with a certain number of terms and a reduced model, in which some of the terms of the full model are removed. The test determines if the increased likelihood of the data using the extra terms in the full model is more than expected if those extra terms are truly zero. Following the Deseq2 guidelines we created three main effect models: time point (1 vs. 3), temperature and line, and included the two main variables that were not analysed in each of these three models as controlling variables. For the time point we also compared the expression patterns between one time point to the two others. We also had three interaction models using all two-way interactions of these three factors. Genes were considered differentially expressed if the adjusted *p*-value was below 0.05. Heatmaps were generated using the rld transformed expression values for DEGs using gplots and Pheatmap implemented in R.

#### Hierarchical clustering analysis and GO enrichment

Clustering of the DEGs was done separately for each tissue. A hierarchical dendrogram was generated using the “hclust” function in R (R v.3.4.4), whereas the “ward. D” objective criterion was used to merge a pair of clusters at each step. Trees were cut at k = 5, k = 3 and k = 3 in hypothalamus, liver and ovary time point models respectively and at k = 3 in hypothalamus for the time point-temperature interaction model to obtain clusters of genes that are expressed the similar way where k is the number of groups. Each cluster’s fold change values at each time point were plotted as profile plots using ggplot2 in R.

For the significant DEGs a GO enrichment analysis was conducted per tissue using the Cytoscape plugin ClueGo 2.5.2 [104] with the human (30.9.2018) gene ontology and KEGG pathway databases [105]. Any transcripts that fell in multiple genes were removed from the analysis. Gene symbols starting with LOC (‘LOC’ + the GeneID is given when published symbol is not available, and orthologs have not yet been officially determined) were investigated by hand to determine if they had an ortholog in other species or if it was non-coding RNA (ncRNA). ClueGo constructs and compares networks of functionally related GO terms with kappa statistics. A two-sided hypergeometric test (enrichment/depletion) was applied with GO term fusion, network specificity and Kappa score were set as default and false discovery correction was carried out using the Bonferroni step-down method.

#### Weighed correlation network and hub gene analysis

The weighed correlation network analysis (WGCNA) [44] was used for getting the co-expression patterns among transcripts and we used the rld transformed data. WGCNA clusters genes with similar patterns of expression across samples to create modules of genes that are likely co-expressed. Because this method uses hierarchical clustering of expression values to group genes into modules, the connectivity of the genes in the modules could reflect the response to time, temperature, line or their interaction. After the modules were created, the correlation of the module eigengenes with time, temperature and line was calculated to examine the strength of the correlation of the module with a given trait. We first removed transcripts with low expression levels across time points (counts smaller than or equal to 4 at one time point) to only have high confidence transcripts and ran the function blockwiseModules to identify potentially co-regulated genes. We created a signed network using soft thresholding power based on the module results of the pickSoftThreshold function (hypothalamus = 6, liver = 20, ovary = 10), minimum module size of 30 transcripts, and a merge cut height of 0.25, 0.45, 0.35 in hypothalamus, liver and ovary, respectively in order to combine the similar modules from same nodes to larger modules (Additional file 28: Figure S11). After this we identified modules that were significantly associated with line, time point and temperature by correlating the module eigengenes with the treatments.

We further analysed the hub genes from the significant modules from each tissue and conducted a STRING pathway analyses [106] in order to see how co-expression translates to functional pathways. Hub genes were defined by module connectivity, measured by the absolute value of the Pearson’s correlation (ModuleMembership > 0.8) and the significance of the relationship with treatments > 0.05. We analysed the hub genes in the STRING plugin (version 1.4.0) in Cytoscape, choosing confidence > 0.4 to construct a protein-protein interaction (PPI) network. In the PPI network, genes with a connectivity degree of ≥10 were also defined as hub genes. The common hub genes both in the co-expression network and the PPI network were regarded as “real” hub genes for subsequent GO enrichments analysis in STRING with default settings. In the PPI network we combined all the tissues together to see how the genes interact together between tissues.

## Additional files

Additional file 1: **Table S1.** Summary of the sequencing and alignment of the three tissue types and 12 pools. (XLSX 11 kb)

Additional file 2: **Table S2.** The number of genes showing significant differential expression in the time point comparisons and interaction models for the three tissues. Time1 is comparison of time point 1 to time point 2 and 3, Time2 is comparison of time point 2 to time point 1 and 3, Time3 is comparison of time point 3 to time point 1 and 2. (XLSX 10 kb)

Additional file 3: **Table S3.** The Likelihood Ratio Test results of the nine models and annotations for the transcripts in hypothalamus. Log2 fold changes are reported in the Log2FC –columns and *P*-values were adjusted for multiple comparisons using Benjamini-Hochberg method. Annotations were based on the *Parus major* annotation release 101 in NCBI. Clusters and modules from the hierarchical clustering analysis and WGCNA are also reported. In the time point main effect model the log2 fold change is between time points 3 vs 1. (XLSX 4991 kb)

Additional file 4: **Table S4.** The Likelihood Ratio Test results of the nine models and annotations for the transcripts in liver. Log2 fold changes are reported in the Log2FC –columns and *P*-values were adjusted for multiple comparisons using Benjamini-Hochberg method. Annotations were based on the *Parus major* annotation release 101 in NCBI. Clusters and modules from the hierarchical clustering analysis and WGCNA are also reported. In the time point main effect model the log2 fold change is between time points 3 vs 1. (XLSX 4342 kb)

Additional file 5: **Table S5.** The Likelihood Ratio Test results of the nine models and annotations for the transcripts in ovary. Log2 fold changes are reported in the Log2FC –columns and P-values were adjusted for multiple comparisons using Benjamini-Hochberg method. Annotations were based on the *Parus major* annotation release 101 in NCBI. Clusters and modules from the hierarchical clustering analysis and WGCNA are also reported. In the time point main effect model the log2 fold change is between time points 3 vs 1. (XLSX 5693 kb)

Additional file 6: **Table S6.** Significant GO terms associated with the time point main effect model gene clusters in hypothalamus. Results based on human GO-database. (XLSX 19 kb)

Additional file 7: **Table S7.** Significant GO terms associated with the time point - temperature interaction model gene clusters in hypothalamus. Results based on human GO-database. (XLSX 120 kb)

Additional file 8: **Table S8.** Significant GO terms associated with the time point main effect model gene clusters in liver. Results based on human GO-database. (XLSX 31 kb)

Additional file 9: **Table S9.** Significant GO terms associated with the time point main effect model gene clusters in ovary. Results based on human GO-database. (XLSX 489 kb)

Additional file 10: **Table S10.** The genes from the super pathway ‘BMAL1-CLOCK, NPAS2 activates circadian gene expression’ found in our time point main effect models and from the time point - temperature interaction model. (XLSX 10 kb)

Additional file 11: **Table S11.** Modules of genes significantly correlated with time, temperature or line in hypothalamus. Gene symbol = annotation, GS = gene significance, p.GS = P-value of gene significance, MMcolor = ModuleMembership correlation coefficient, p.MMcolor = ModuleMembership *p*-value. (XLSX 4497 kb)

Additional file 12: **Table S12.** Modules of genes significantly correlated with time, temperature or line in liver. Gene symbol = annotation, GS = gene significance, p.GS = P-value of gene significance, MMcolor = ModuleMembership correlation coefficient, p.MMcolor = ModuleMembership p-value. (XLSX 5375 kb)

Additional file 13: **Table S13.** Modules of genes significantly correlated with time, temperature or line in ovary. Gene symbol = annotation, GS = gene significance, p.GS = P-value of gene significance, MMcolor = ModuleMembership correlation coefficient, p.MMcolor = ModuleMembership p-value. (XLSX 6338 kb)

Additional file 14: **Table S14.** List of highly connected module genes in hypothalamus that have at least one connection degree in the PPI network. (XLSX 42 kb)

Additional file 15: **Table S15.** List of highly connected module genes in liver that have at least one connection degree in the PPI network. (XLSX 13 kb)

Additional file 16: **Table S16.** List of highly connected module genes in ovary that have at least one connection degree in the PPI network. (XLSX 100 kb)

Additional file 17: **Table S17.** Significant GO terms associated with the real hub genes. Results based on human GO-database. (XLSX 18 kb)

Additional file 18: **Figure S1.** Clustering of samples based on principal component analysis (PCA). Samples collected from warm (W) and cold (C) temperature treatments from two different lines, early (E) and late (L), from three different time points and from three different tissues: a. hypothalamus, b. liver and c. ovary. (PDF 102 kb)

Additional file 19: **Figure S2.** Volcano plots of all the transcripts analysed in hypothalamus RNA-seq in six different models. Genes differentially expressed with *p* < 0.05 after correcting for false discovery rate are in orange. Genes with a *p* > 0.05 after correcting for false discovery rate are in black. (PDF 20 kb)

Additional file 20: **Figure S3.** Volcano plots of all the transcripts analysed in liver RNA-seq in six different models. Genes differentially expressed with p < 0.05 after correcting for false discovery rate are in orange. Genes with a p > 0.05 after correcting for false discovery rate are in black. (PDF 19 kb)

Additional file 21: **Figure S4.** Volcano plots of all the transcripts analysed in ovary RNA-seq in six different models. Genes differentially expressed with p < 0.05 after correcting for false discovery rate are in orange. Genes with a p > 0.05 after correcting for false discovery rate are in black. (PDF 20 kb)

Additional file 22: **Figure S5.** Expression patterns of DEG clusters in hypothalamus time point main effect model. (PDF 31 kb)

Additional file 23: **Figure S6.** Expression patterns of DEG clusters in hypothalamus time point-temperature interaction model. (PDF 144 kb)

Additional file 24: **Figure S7.** Expression patterns of DEG clusters in liver time point main effect model. (PDF 22 kb)

Additional file 25: **Figure S8.** Expression patterns of DEG clusters in ovary time point main effect model. (PDF 72 kb)

Additional file 26: **Figure S9.** The raw expression levels of *ZP4* in ovary. Numbers indicate different time points from the study. (PDF 8 kb)

Additional file 27: **Figure S10.** The raw expression levels of *CTSEAL*, *VTG2* (VTG2a - LOC107208431and VTG2b - LOC107208432) and *APOV1* in liver. (PDF 12 kb)

Additional file 28: **Figure S11.** Hierarchical clustering tree based on WGCNA module eigengenes in A. hypothalamus, B. liver and C. ovary. (PDF 105 kb)

Additional file 29: **Figure S12.** Matrix with the module-treatment relationships and corresponding *p*-values between the detected modules on the y-axis and treatments on the x-axis based on hypothalamus RNA-seq. The relationships are coloured based on their correlation: red is a strong positive correlation, while blue is a strong negative correlation. The value at the top of each square represents the correlation coefficient between the module eigengene and the treatment with the correlation p-value in parentheses. (PDF 42 kb)

Additional file 30: **Figure S13.** Matrix with the module-treatment relationships and corresponding p-values between the detected modules on the y-axis and treatments on the x-axis based on liver RNA-seq. The relationships are coloured based on their correlation: red is a strong positive correlation, while blue is a strong negative correlation. The value at the top of each square represents the correlation coefficient between the module eigengene and the treatment with the correlation p-value in parentheses. (PDF 51 kb)

Additional file 31: **Figure S14.** Matrix with the module-treatment relationships and corresponding p-values between the detected modules on the y-axis and treatments on the x-axis based on ovary RNA-seq. The relationships are coloured based on their correlation: red is a strong positive correlation, while blue is a strong negative correlation. The value at the top of each square represents the correlation coefficient between the module eigengene and the treatment with the correlation p-value in parentheses. (PDF 44 kb)

Additional file 32: **Figure S15.** Daily minimum (A) and daily maximum (B) temperatures for the cold (blue) and warm (red) spring provided in the first and second breeding season. The open triangle indicates the day on which the first breeding season stopped and birds went into the phase of the experiment where days were shortened and the temperature set at 10 °C (see ‘Second breeding season’) in to prepare them for the second breeding season. The black triangles indicate the three time points (66 January = 7 March, 89 January = March 30, 110 January = April 20) on which the birds were sacrificed in the second breeding season. (PDF 9 kb)

## Data Availability

Sequence data: All quality-trimmed reads used in this study are available for download at the Sequence Read Archive (accession numbers SRR9644032-SRR9644067, bioproject PRJNA208335).

## Abbreviations

DEG: Differentially expressed genes
HPGL: Hypothalamic-pituitary-gonadal-liver
LRT: Likelihood Ratio Test
PCA: Principal Component Analysis
rld: Regularized log transformation procedure
WGCNA: Weighed correlation network analysis
