# Supplementary material for: Exploration of tissue-specific gene expression patterns underlying timing of breeding in contrasting temperature environments in a song bird
Source: BMC Genomics. 2019 Sep 2;20:693. doi: 10.1186/s12864-019-6043-0 (PMC6720064; doi:10.1186/s12864-019-6043-0)
Supplement: Supplementary file 18 — Figure S1. Clustering of samples based on principal component analysis (PCA). Samples collected from warm (W) and cold (C) temperature treatments from two different lines, early (E) and late (L), from three different time points and from three different tissues: a. hypothalamus, b. liver and c. ovary. (PDF 102 kb) [file 12864_2019_6043_MOESM18_ESM.pdf]

A

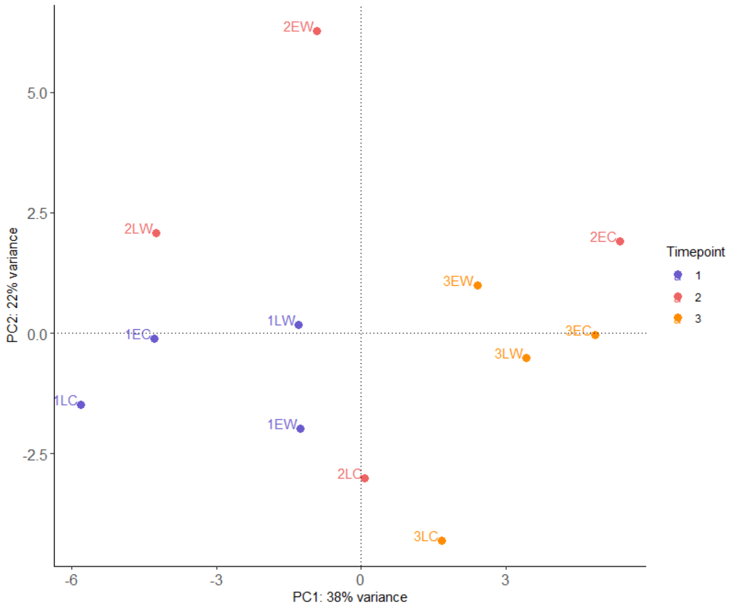

B

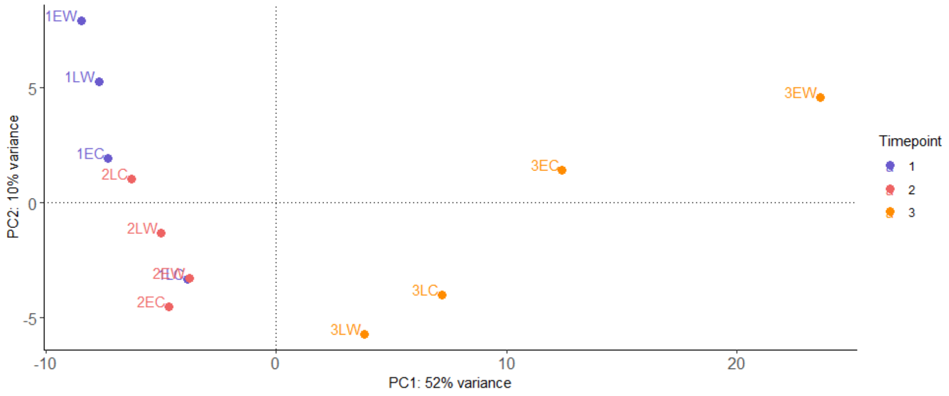

C

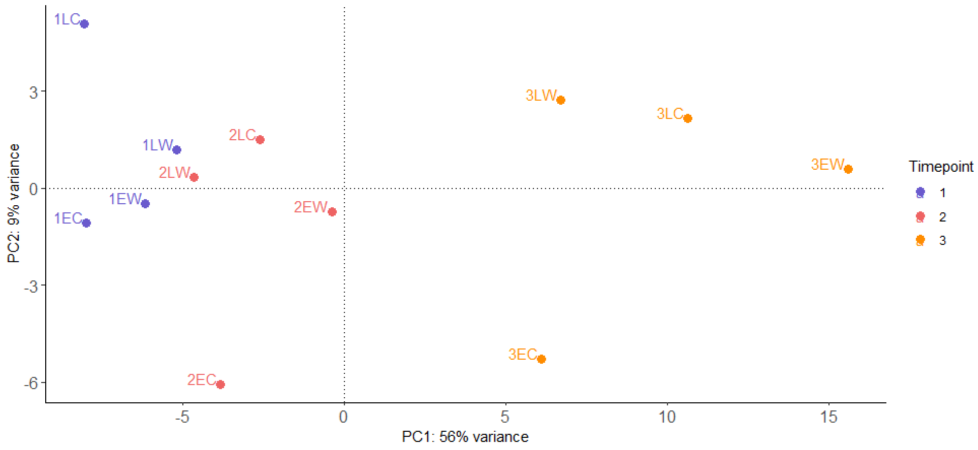

Fig S1. Clustering of samples based on principal component analysis (PCA). Samples collected from warm (W) and cold (C) temperature treatments from two different lines, early (E) and late (L), from three different time points and from three different tissues: a. hypothalamus, b. liver and c. ovary.
