# Supplementary material for: Exploration of tissue-specific gene expression patterns underlying timing of breeding in contrasting temperature environments in a song bird
Source: BMC Genomics. 2019 Sep 2;20:693. doi: 10.1186/s12864-019-6043-0 (PMC6720064; doi:10.1186/s12864-019-6043-0)
Supplement: Supplementary file 20 — Figure S3. Volcano plots of all the transcripts analysed in liver RNA-seq in six different models. Genes differentially expressed with p < 0.05 after correcting for false discovery rate are in orange. Genes with a p > 0.05 after correcting for false discovery rate are in black. (PDF 19 kb) [file 12864_2019_6043_MOESM20_ESM.pdf]

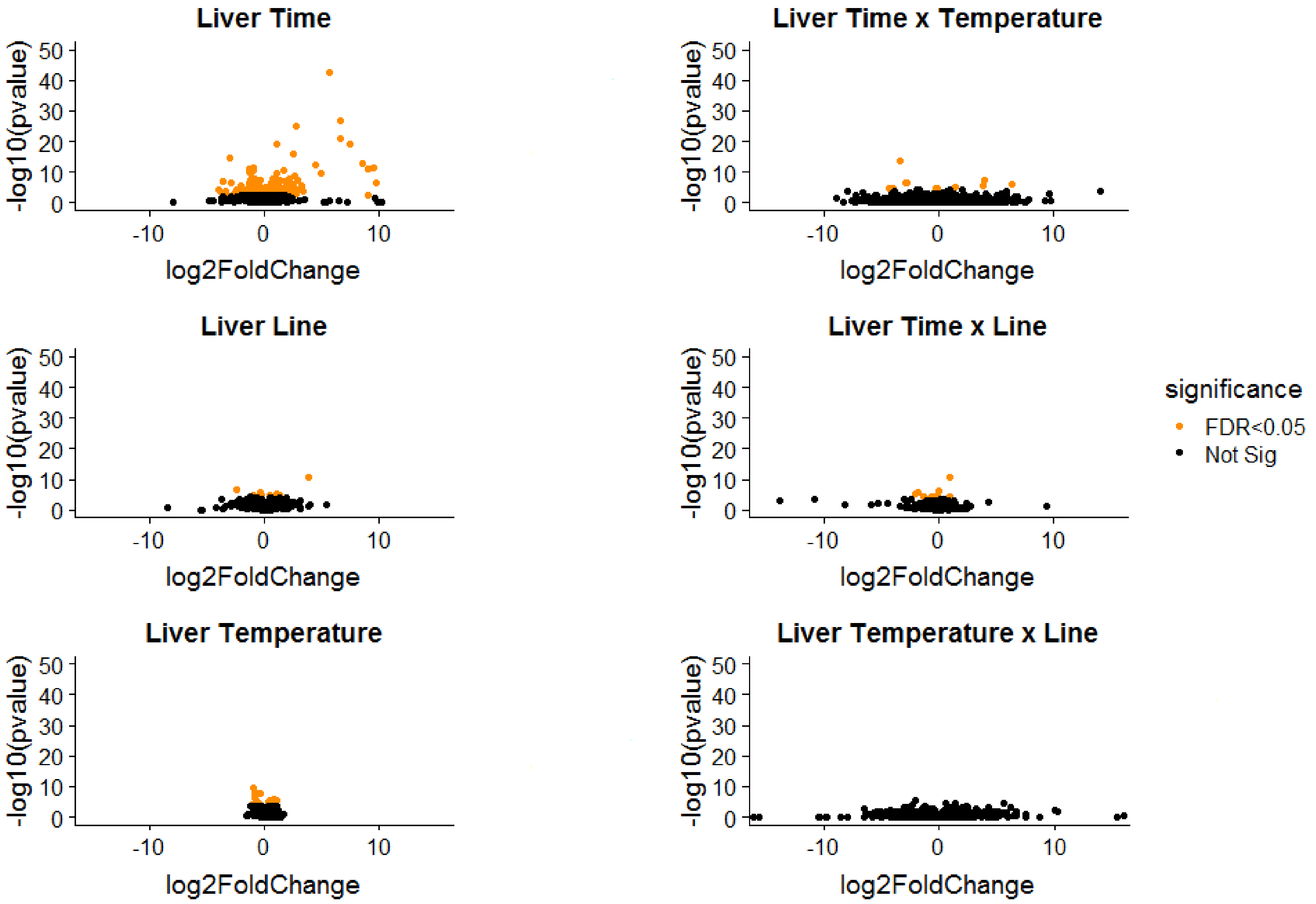

Fig S3. Volcano plots of all the transcripts analysed in liver RNA-seq in six different models. Genes differentially expressed with $p < 0.05$ after correcting for false discovery rate are in orange. Genes with a $p > 0.05$ after correcting for false discovery rate are in black.
