# Supplementary figures and images for: Exploration of tissue-specific gene expression patterns underlying timing of breeding in contrasting temperature environments in a song bird
Source: BMC Genomics. 2019 Sep 2;20:693. doi: 10.1186/s12864-019-6043-0 (PMC6720064; doi:10.1186/s12864-019-6043-0)

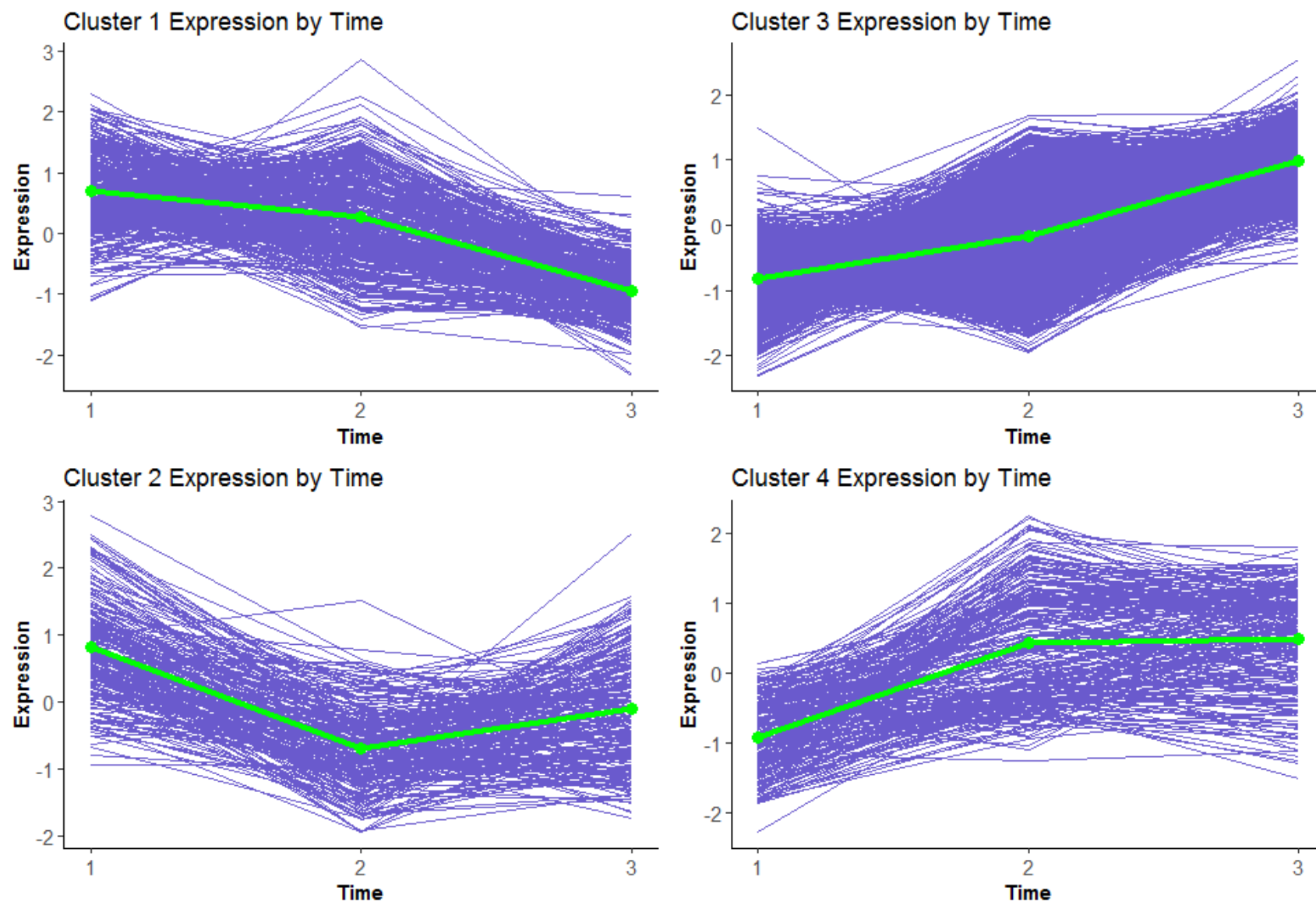

Fig S5. Expression patterns of DEG clusters in hypothalamus time point main effect model.

Supplement: Supplementary file 22 — Figure S5. Expression patterns of DEG clusters in hypothalamus time point main effect model. (PDF 31 kb) [file 12864_2019_6043_MOESM22_ESM.pdf]

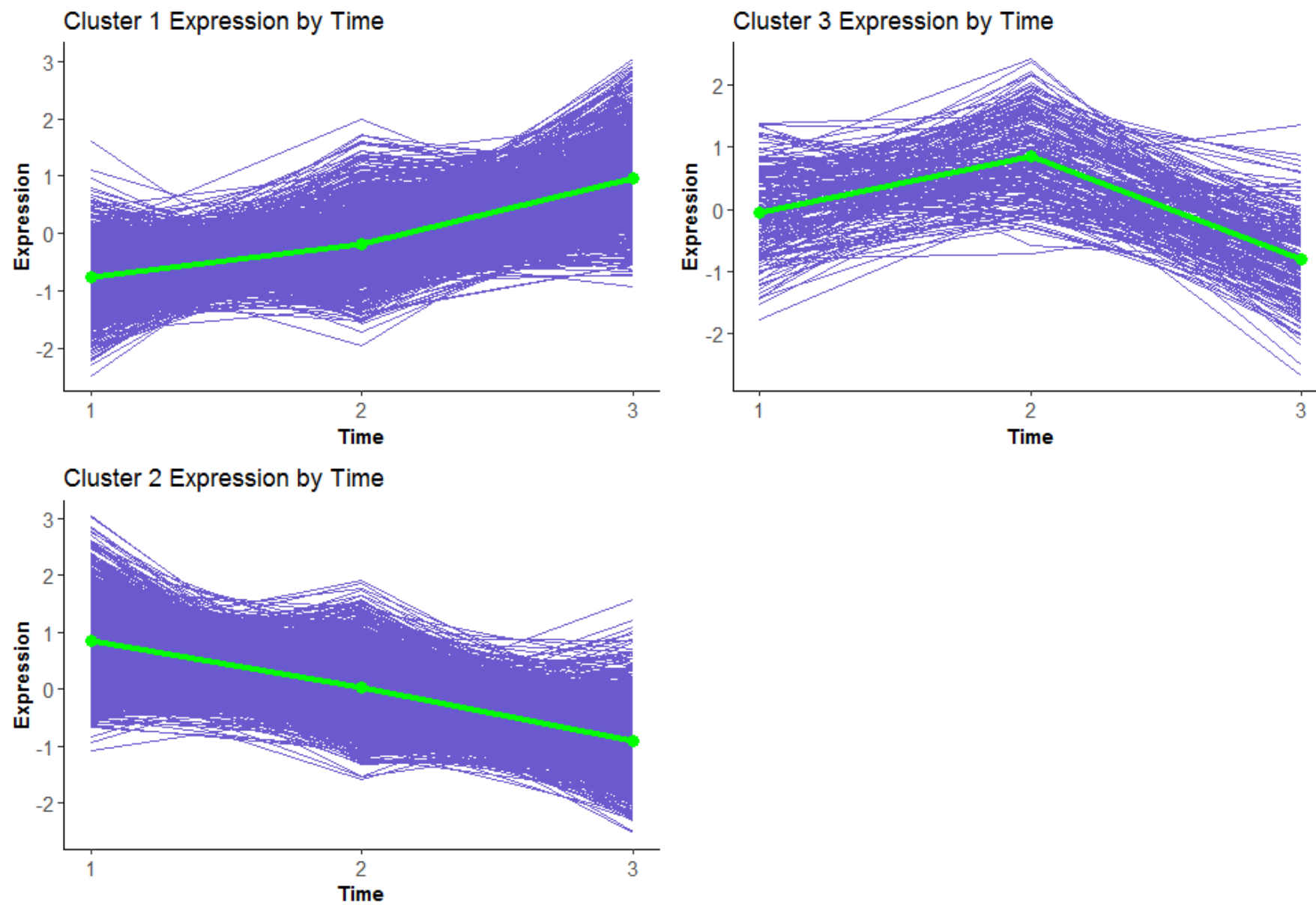

Fig S7. Expression patterns of DEG clusters in liver time point main effect model.

Supplement: Supplementary file 24 — Figure S7. Expression patterns of DEG clusters in liver time point main effect model. (PDF 22 kb) [file 12864_2019_6043_MOESM24_ESM.pdf]

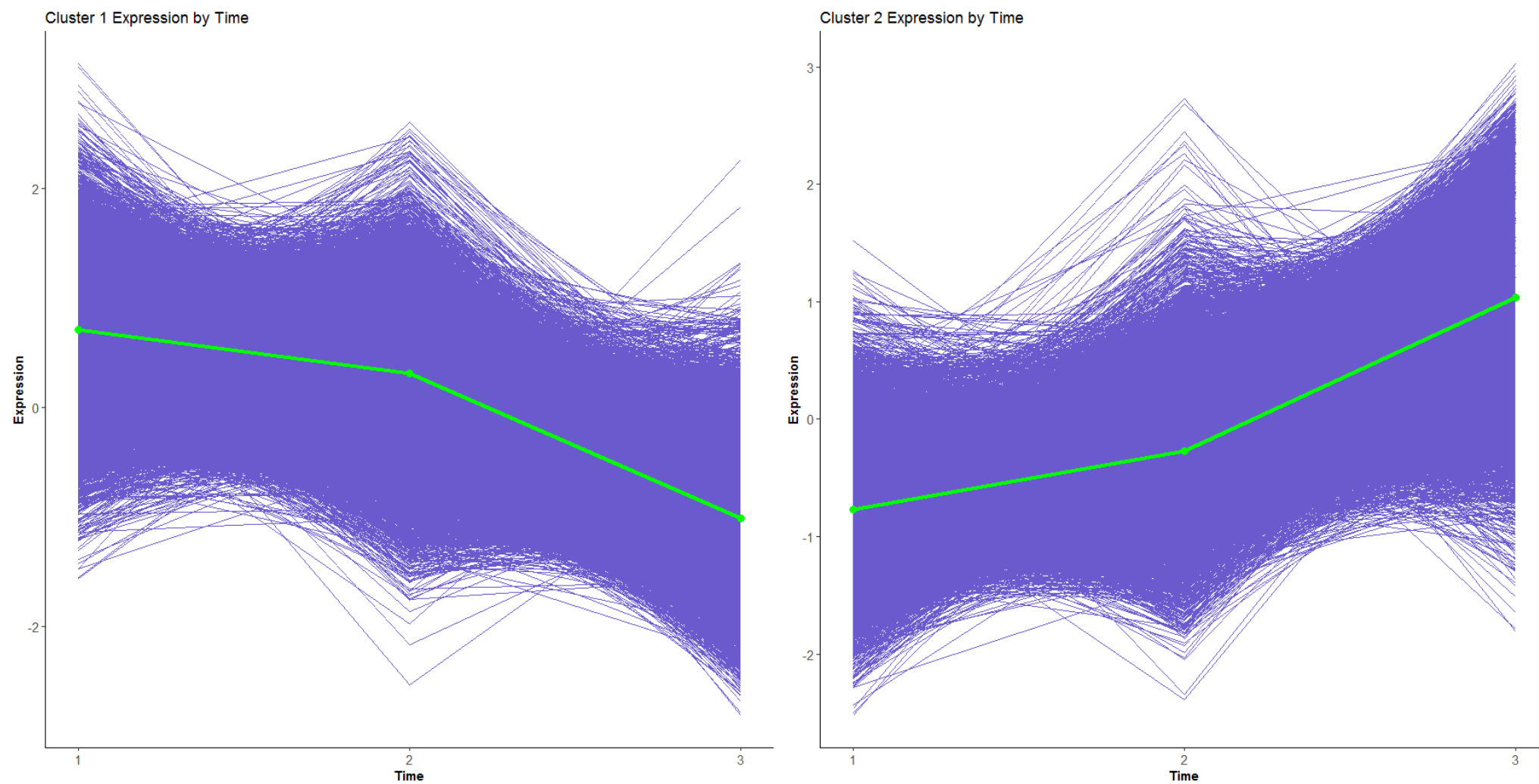

Fig S8. Expression patterns of DEG clusters in ovary time point main effect model.

Supplement: Supplementary file 25 — Figure S8. Expression patterns of DEG clusters in ovary time point main effect model. (PDF 72 kb) [file 12864_2019_6043_MOESM25_ESM.pdf]

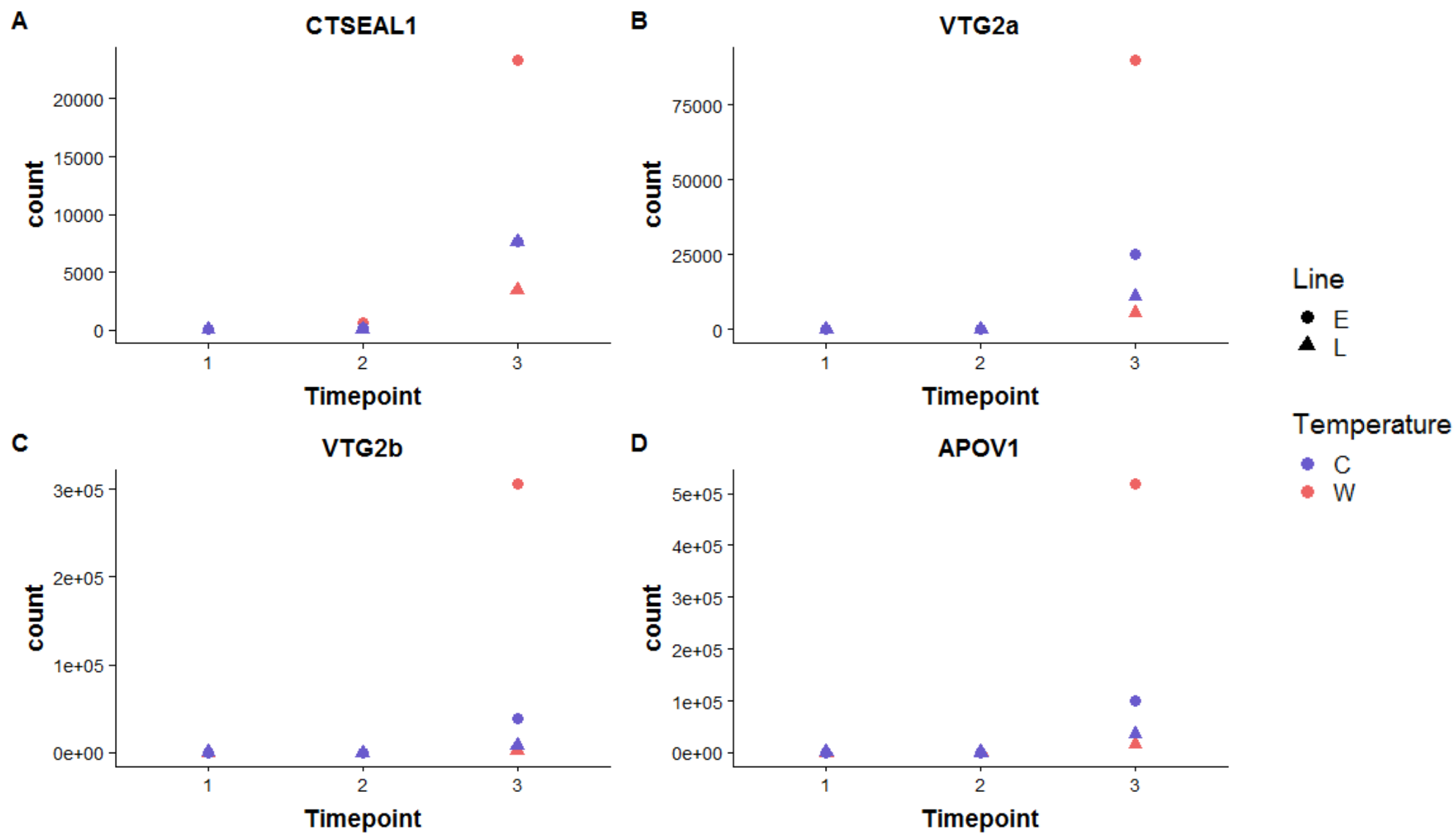

Fig S10. The raw expression levels of *CTSEAL*, *VTG2* (*VTG2a* - LOC107208431 and *VTG2b* - LOC107208432) and *APOV1* in liver.

Supplement: Supplementary file 27 — Figure S10. The raw expression levels of CTSEAL, VTG2 (VTG2a - LOC107208431and VTG2b - LOC107208432) and APOV1 in liver. (PDF 12 kb) [file 12864_2019_6043_MOESM27_ESM.pdf]

Daily minimum temperature (°C)

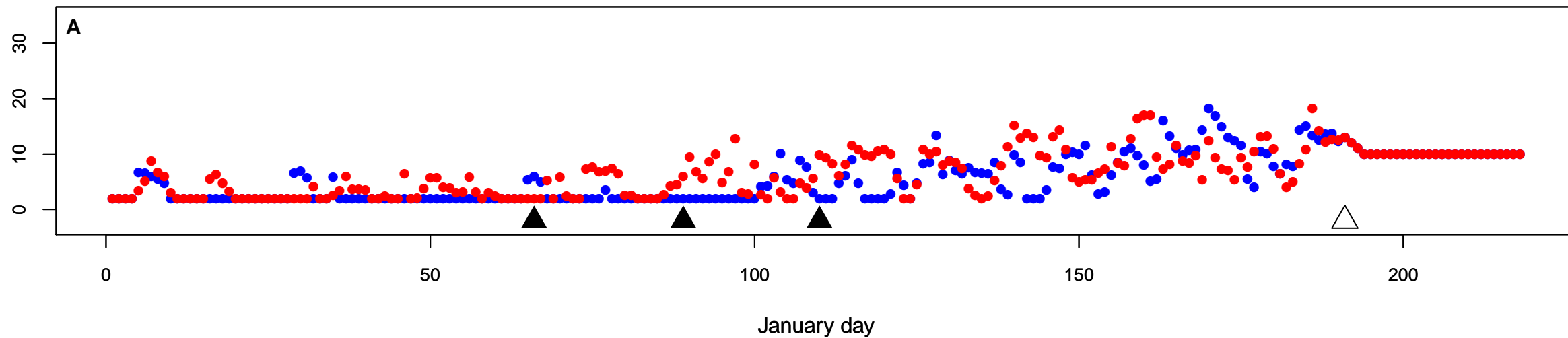

Daily maximum temperature (°C)

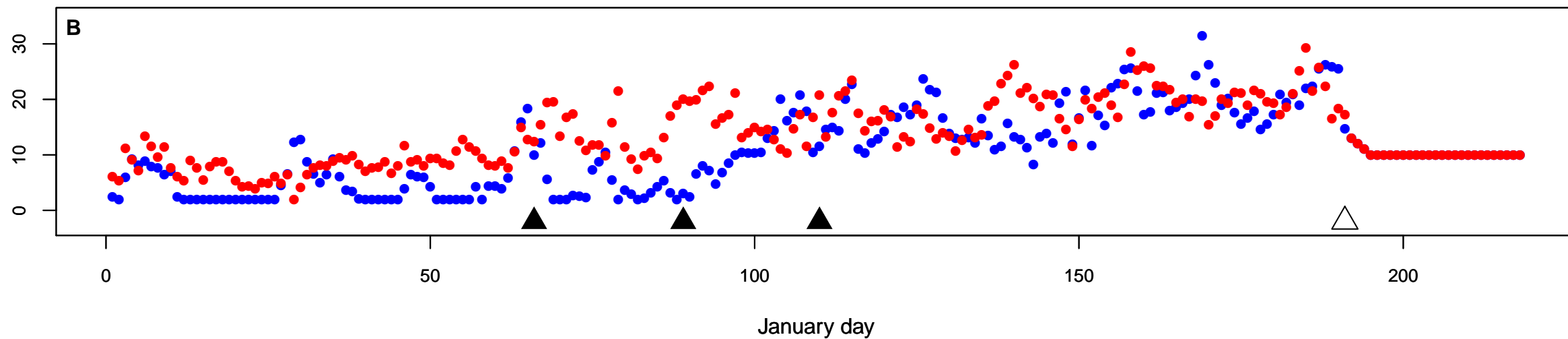

Supplement: Supplementary file 32 — Figure S15. Daily minimum (A) and daily maximum (B) temperatures for the cold (blue) and warm (red) spring provided in the first and second breeding season. The open triangle indicates the day on which the first breeding season stopped and birds went into the phase of the experiment where days were shortened and the temperature set at 10 °C (see ‘Second breeding season’) in to prepare them for the second breeding season. The black triangles indicate the three time points (66 January = 7 March, 89 January = March 30, 110 January = April 20) on which the birds were sacrificed in the second breeding season. (PDF 9 kb) [file 12864_2019_6043_MOESM32_ESM.pdf]
