# Supplementary material for: Exploration of tissue-specific gene expression patterns underlying timing of breeding in contrasting temperature environments in a song bird
Source: BMC Genomics. 2019 Sep 2;20:693. doi: 10.1186/s12864-019-6043-0 (PMC6720064; doi:10.1186/s12864-019-6043-0)
Supplement: Supplementary file 23 — Figure S6. Expression patterns of DEG clusters in hypothalamus time point-temperature interaction model. (PDF 144 kb) [file 12864_2019_6043_MOESM23_ESM.pdf]

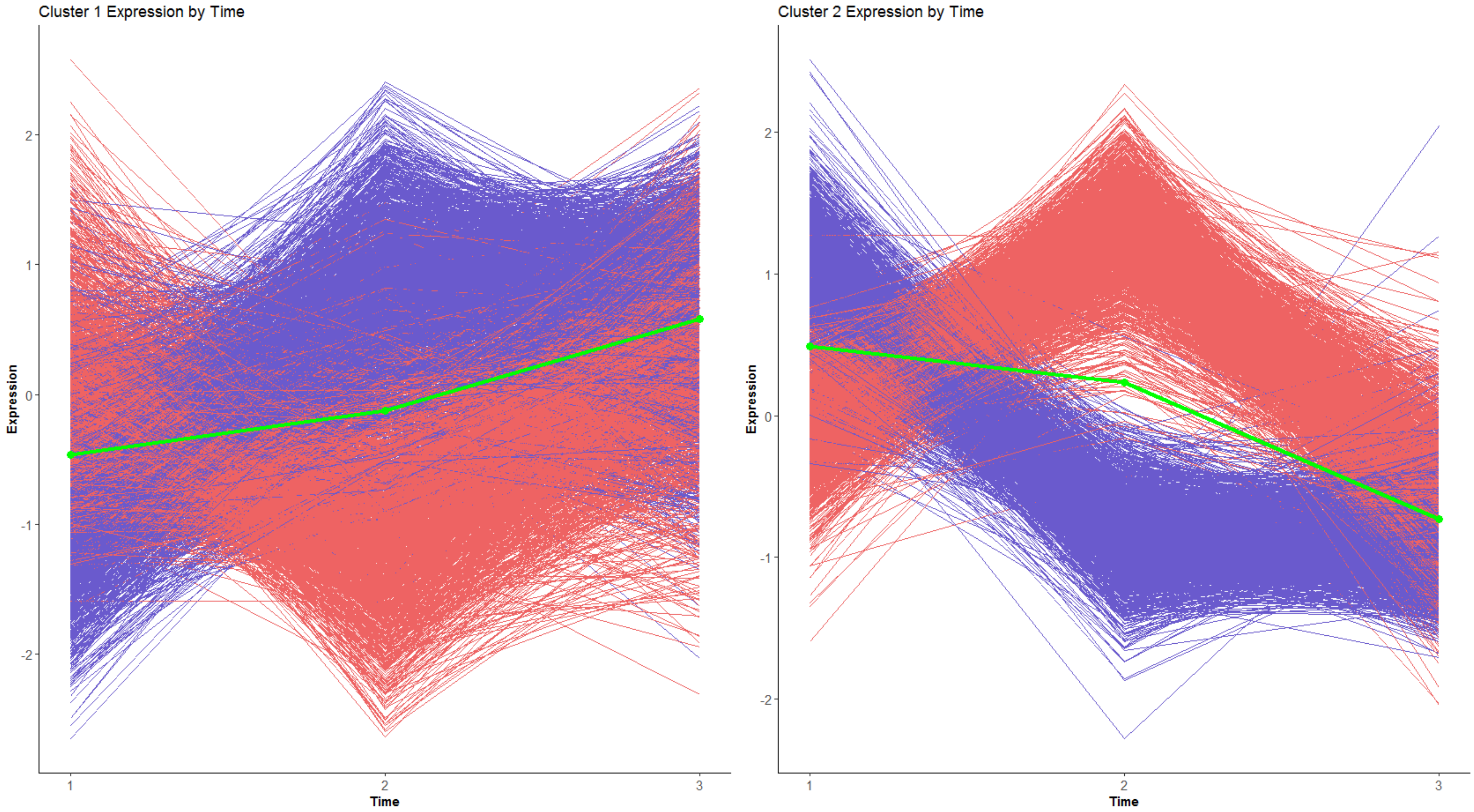

Fig S6. Expression patterns of DEG clusters in hypothalamus time point-temperature interaction model.
