# Supplementary material for: Exploration of tissue-specific gene expression patterns underlying timing of breeding in contrasting temperature environments in a song bird
Source: BMC Genomics. 2019 Sep 2;20:693. doi: 10.1186/s12864-019-6043-0 (PMC6720064; doi:10.1186/s12864-019-6043-0)
Supplement: Supplementary file 26 — Figure S9. The raw expression levels of ZP4 in ovary. Numbers indicate different time points from the study. (PDF 8 kb) [file 12864_2019_6043_MOESM26_ESM.pdf]

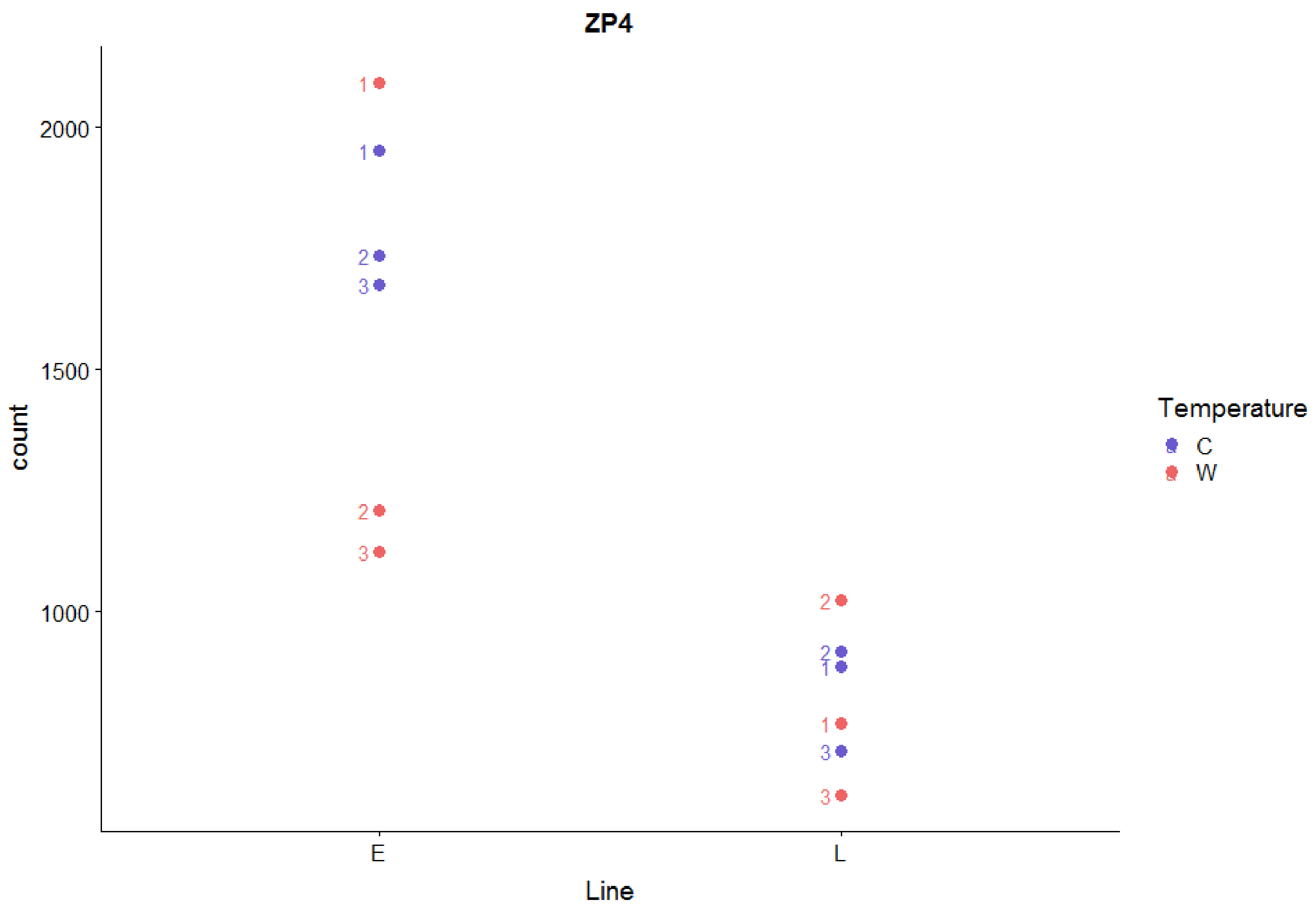

Fig S9. The raw expression levels of *ZP4* in ovary. Numbers indicate different time points from the study.
