# Supplementary material for: Exploration of tissue-specific gene expression patterns underlying timing of breeding in contrasting temperature environments in a song bird
Source: BMC Genomics. 2019 Sep 2;20:693. doi: 10.1186/s12864-019-6043-0 (PMC6720064; doi:10.1186/s12864-019-6043-0)
Supplement: Supplementary file 28 — Figure S11. Hierarchical clustering tree based on WGCNA module eigengenes in A. hypothalamus, B. liver and C. ovary. (PDF 105 kb) [file 12864_2019_6043_MOESM28_ESM.pdf]

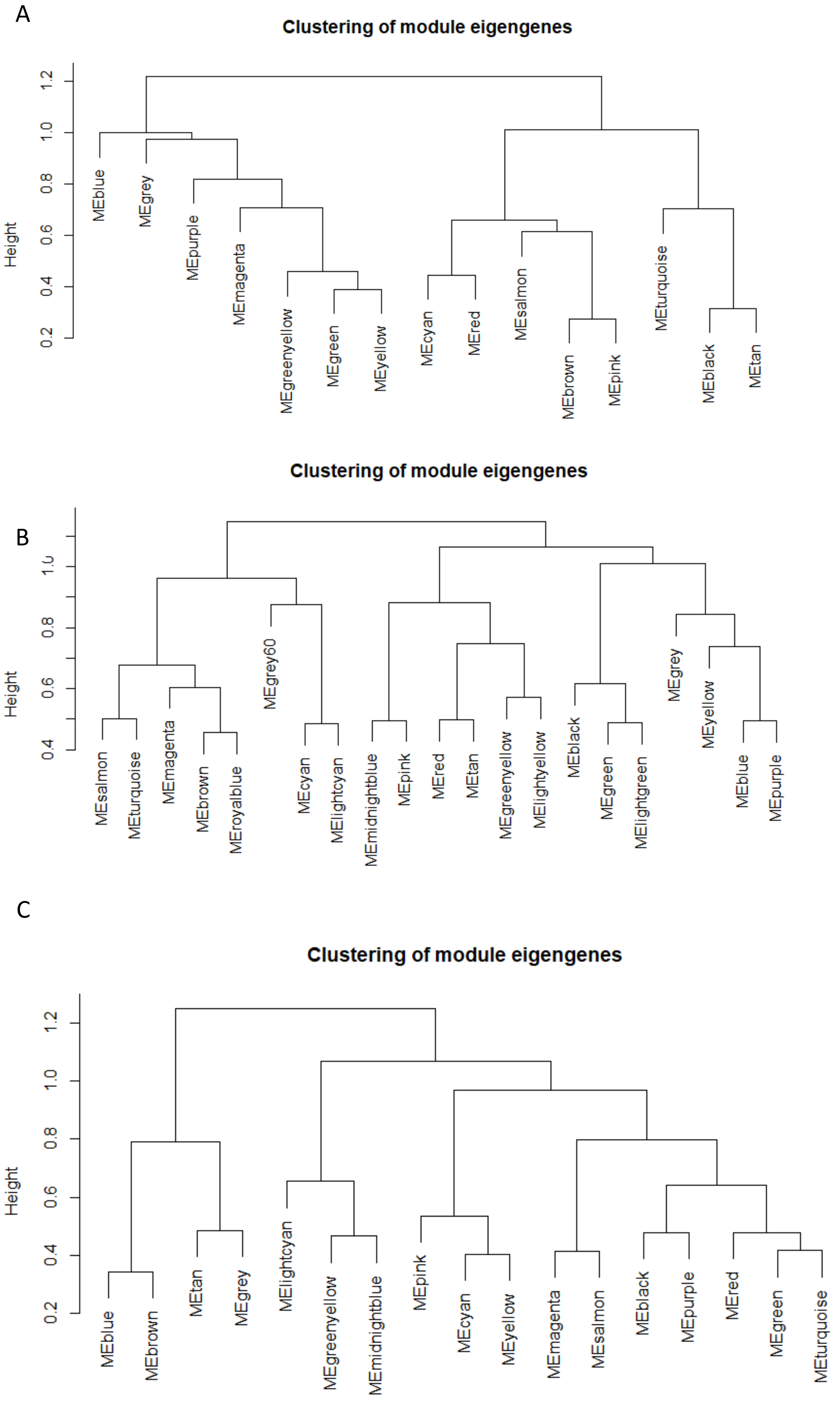

Fig S11. Hierarchical clustering tree based on WGCNA module eigengenes in A. hypothalamus, B. liver and C. ovary
