# Supplementary material for: Exploration of tissue-specific gene expression patterns underlying timing of breeding in contrasting temperature environments in a song bird
Source: BMC Genomics. 2019 Sep 2;20:693. doi: 10.1186/s12864-019-6043-0 (PMC6720064; doi:10.1186/s12864-019-6043-0)
Supplement: Supplementary file 31 — Figure S14. Matrix with the module-treatment relationships and corresponding p-values between the detected modules on the y-axis and treatments on the x-axis based on ovary RNA-seq. The relationships are coloured based on their correlation: red is a strong positive correlation, while blue is a strong negative correlation. The value at the top of each square represents the correlation coefficient between the module eigengene and the treatment with the correlation p-value in parentheses. (PDF 44 kb) [file 12864_2019_6043_MOESM31_ESM.pdf]

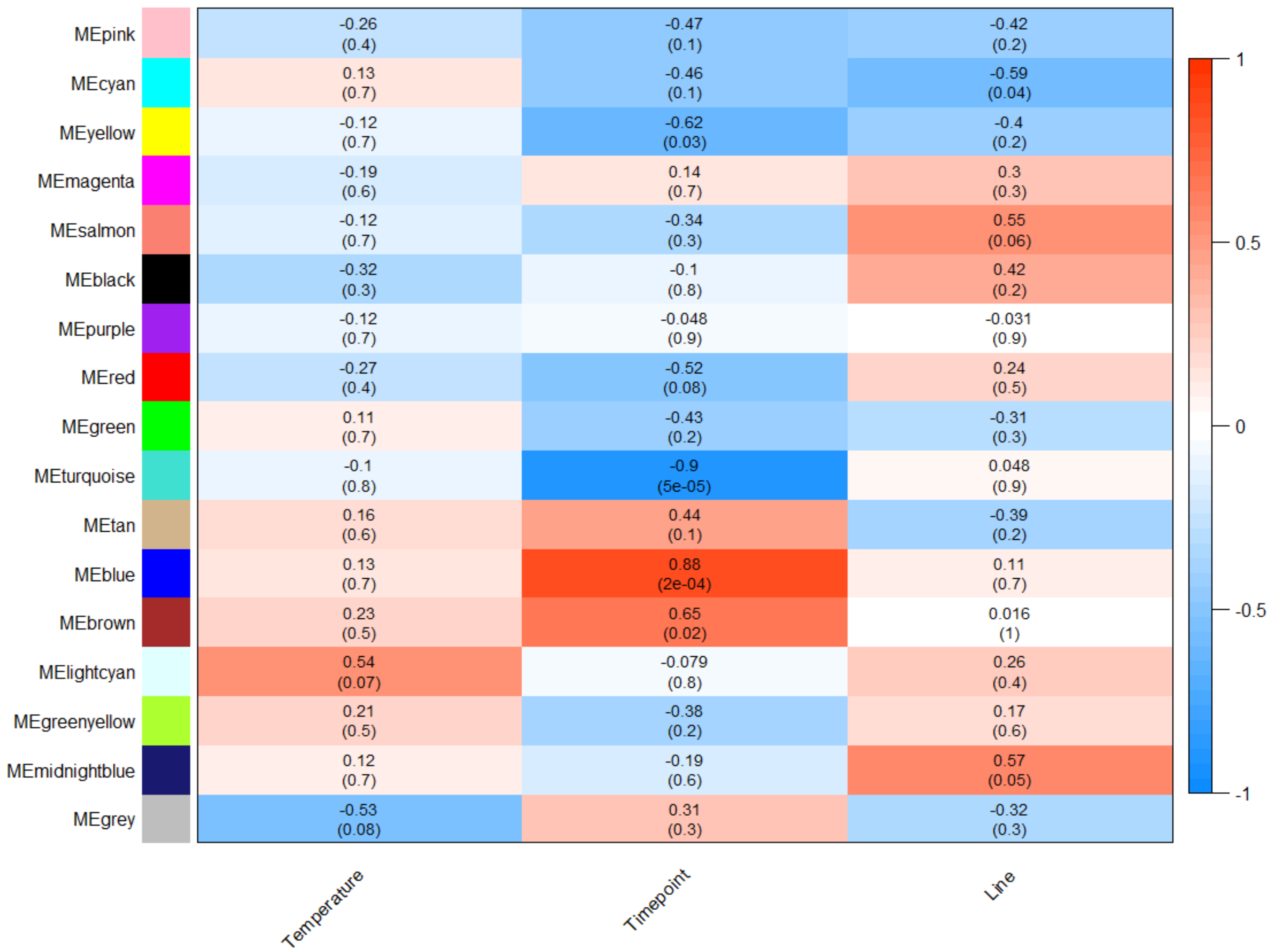

Fig S14. Matrix with the module-treatment relationships and corresponding p-values between the detected modules on the y-axis and treatments on the x-axis based on ovary RNA-seq. The relationships are coloured based on their correlation: red is a strong positive correlation, while blue is a strong negative correlation. The value at the top of each square represents the correlation coefficient between the module eigengene and the treatment with the correlation p-value in parentheses.
